# Overexpression of *VIRE*2-*INTERACTING PROTEIN*2 in Arabidopsis regulates genes involved in *Agrobacterium*-mediated plant transformation and abiotic stresses

**DOI:** 10.1038/s41598-019-49590-3

**Published:** 2019-09-18

**Authors:** Vidhyavathi Raman, Ajith Anand, Balaji Vasudevan, Mustafa R. Morsy, Bikram D. Pant, Hee-Kyung Lee, Yuhong Tang, Kirankumar S. Mysore

**Affiliations:** 1 Noble Research Institute, LLC., Ardmore, Oklahoma USA; 2 Present Address: Corteva Agriscience, Johnston, Iowa 50131 USA; 3 Present Address: GreenLight Biosciences, Inc., Durham, North Carolina USA; 4 Present Address: The Department of Biological and Environmental Sciences, UWA, Station 7, Livingston, Alabama 35470 USA

**Keywords:** Molecular engineering in plants, Abiotic

## Abstract

Arabidopsis VIRE2-INTERACTING PROTEIN2 (VIP2) was previously described as a protein with a NOT domain, and Arabidopsis *vip2* mutants are recalcitrant to *Agrobacterium*-mediated root transformation. Here we show that VIP2 is a transcription regulator and the C-terminal NOT2 domain of VIP2 interacts with VirE2. Interestingly, *AtVIP2* overexpressor lines in Arabidopsis did not show an improvement in *Agrobacterium*-mediated stable root transformation, but the transcriptome analysis identified 1,634 differentially expressed genes compared to wild-type. These differentially expressed genes belonged to various functional categories such as membrane proteins, circadian rhythm, signaling, response to stimulus, regulation of plant hypersensitive response, sequence-specific DNA binding transcription factor activity and transcription regulatory region binding. In addition to regulating genes involved in *Agrobacterium*-mediated plant transformation, *AtVIP2* overexpressor line showed differential expression of genes involved in abiotic stresses. The majority of the genes involved in abscisic acid (ABA) response pathway, containing the Abscisic Acid Responsive Element (ABRE) element within their promoters, were down-regulated in *AtVIP2* overexpressor lines. Consistent with this observation, *AtVIP2* overexpressor lines were more susceptible to ABA and other abiotic stresses. Based on the above findings, we hypothesize that VIP2 not only plays a role in *Agrobacterium*-mediated plant transformation but also acts as a general transcriptional regulator in plants.

## Introduction

The plant pathogen *Agrobacterium tumefaciens* causes neoplastic growth called crown galls on plants by transferring genetic material coded on the transfer DNA (T-DNA) from the tumor-inducing (Ti plasmid) to plant cells, resulting in the genome modification following integration (see reviews^1–4^). The genetic transformation of a plant cell by *A*. *tumefaciens* involves the synthesis and translocation of the T-DNA mediated by the virulence (*vir*) gene products, interaction of the translocated virulence proteins with their cognate partners in the host, and expression of the genes on the T-DNA following integration (see reviews^1,2,5,6^). Some of the virulence proteins, namely VirD2, VirD5, VirE2, VirE3, and VirF, are *A*. *tumefaciens* effector proteins that are directly translocated into the plant cell via the type IV secretion system (T4SS)^7,8^.

One of the translocated virulence proteins, VirE2, binds to the single-stranded DNA (ssDNA), *in vitro*, to form a telephone-cord like structure protecting it from degradation by nucleases^9–12^. Plant proteins that interact with VirE2 were surveyed by the yeast two-hybrid system, resulting in the identification of two VirE2-interacting proteins (VIP), VIP1 and VIP2^13^. VirE2 is suggested to piggy-back on VIP1 for nuclear import via an importin α-dependent pathway and this process is up-regulated by a host MAP kinase that phosphorylates VIP1^14^. VIP2 is a negative on TATA-less (NOT)-domain containing protein which likely functions as a transcription factor and is required for plant stable transformation, but not for transient T-DNA expression^15^. An Arabidopsis *vip2* mutant was shown to be deficient in T-DNA integration due to changes in the expression of many genes, including core histones, suggesting that VIP2 has a role in T-DNA integration^15^.

A NOT2 homolog in yeast acts as a general negative regulator of gene expression. Similarly, in Drosophila, a NOT2 homolog, the Rga protein, has probable function in mediating interaction between chromatin proteins and the transcriptional complex^16,17^. NOT2 is the core member of the CCR4-NOT complex that regulates mRNA metabolism at both transcriptional and posttranscriptional levels^18^ and has a role in promoting transcriptional elongation by RNA polymerase II^19,20^. The NOT proteins (VIP2/NOT2b and NOT2a) are general transcriptional regulators essential for plant development. NOT2s acts as a scaffold to interact with RNA polymerase II, and promotes transcription of both protein coding and miRNA genes, and facilitates efficient DICER-LIKE1 recruitment in miRNA biogenesis^21^. VIP2/NOT2b interact with miRNA processing factors such as cap binding proteins, CBP80 and CBP20^21^, and the interaction is modulated by VirD5^22^.

Here, we demonstrate that VIP2 is a transcription regulator and overexpression of VIP2 in Arabidopsis modulates expression of genes not only involved in *Agrobacterium*-mediated plant transformation but also genes associated with several other pathways including abiotic stresses. This finding is consistent with its role as transcription regulator. Interestingly, overexpression of *VIP2* in Arabidopsis did not significantly increase *Agrobacterium*-mediated root transformation efficiency.

## Results

### VIP2 is a transcriptional regulator

**Figure 1:**
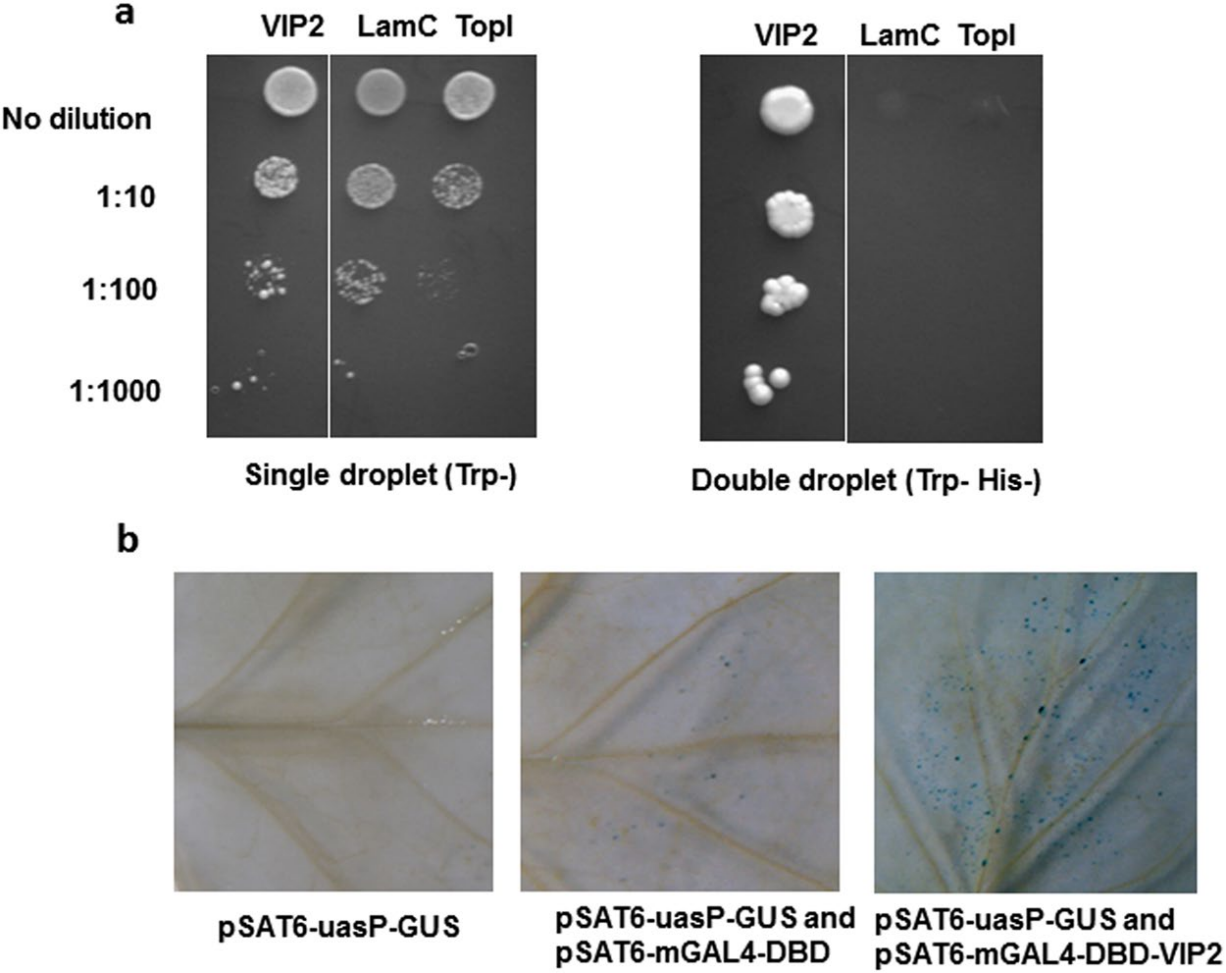
Yeast one-hybrid assays and *in planta* transactivation assays suggest VIP2 is a putative transcription regulator. (**a**) Single and double dropout assays were carried out on SD medium lacking Trp, or Trp and His respectively, and containing 10 mM 3-AT. Undiluted, and dilutions of 1:10, 1:100 and 1:1000 were plated. The experiments were repeated twice. (**b**) A constitutive *CaMV*35*S* promoter driving *VIP2* was fused to the DNA binding domain (DBD) of a GAL4/UAS transactivation expression system in combination with the GUS reporter protein. GUS histochemical staining of tobacco leaves bombarded with different reporter constructs *pSAT6*-*uasP*-*GUS* only, *pSAT6*-*uasP*-*GUS* and *pSAT6*-*mGAL4*-*DBD* constructs, and *pSAT6*-*uasP*-*GUS* and *pSAT6*-*mGAL4*-*DBD*-*VIP2* constructs were carried out. The experiments were repeated three times with similar results.

Based on the VIP2 sequence, transcript profiling data of Arabidopsis *vip2* mutant^15^, and nuclear localization data, we hypothesized that VIP2 is a transcriptional regulator. To explore this hypothesis, we first tested whether VIP2 can activate transcription in yeast when bound to DNA as previously described for VirE3^23^. We therefore tested the VIP2 protein for transcriptional activation through yeast one-hybrid assay^24^. For this purpose, the VIP2 (GenBank # AF295433) open reading frame was fused to the GAL4 DNA binding domain (DBD) and cloned into a *pGBKT7* yeast vector. The human lamin C (LamC) and topoisomerase I (TopI) genes cloned into the *pGBKT7* vector were used as controls. We performed single and double dropout assays to confirm the DNA binding ability of the VIP2 protein in yeast. Undiluted and a 1:10 dilution of all the four clones were able to grow well on solid synthetic dropout (SD) media lacking tryptophan. However, 1:100 and 1:1000 dilutions showed little to no growth (Fig. 1a). The VIP2 fusion construct grew on media lacking Trp and His and containing 10 mM 3-amino-1,2,4-triazole (3-AT), while the LamC and TopI did not (Fig. 1a). These results suggest that the VIP2 protein can activate and express the *his3* gene, allowing yeast to grow on a histidine and tryptophan-deficient medium. The above findings suggest VIP2 can activate transcription in yeast, and therefore potentially is a transcription regulator.

To validate the transcription activation property of VIP2 *in planta*, a promoter trans-activation assay, as described earlier^25^, was used. Tobacco leaves were bombarded with different combinations of reporter constructs such as *pSAT6*-*uasP*-*GUS* only, *pSAT6*-*uasP*-*GUS* and *pSAT6*-*mGAL4*-*DBD* constructs and, *pSAT6*-*uasP*-*GUS* and *pSAT6*-*mGAL4*-*DBD*-*VIP2* constructs (Fig. 1b). GUS histochemical staining was performed 48–72 h post DNA bombardment, and the following observations were recorded. We observed few blue spots in the controls, *pSAT6*-*uasP*-*GUS* alone (32 ± 8 blue spots) or *pSAT6*-*uasP*-*GUS* vector expressing the unfused GAL4 DBD (*pSAT6*-*mGAL4*-*DBD*; 69 ± 12 blue spots) and weak expression of the GUS reporter (Fig. 1b). In contrast, co-bombardment of *pSAT6*-*uasP*-*GUS* with *pSAT6*-*mGAL4*-*DBD*-*VIP2* that expresses *mGAL4*-*VIP2* fusion from the *CaMV35S* promoter resulted in a significantly higher number of blue spots (685 ± 181 blue spots) and increased expression of the GUS reporter (Fig. 1b). Based on the above results we hypothesized that VIP2 is efficient in transactivation of the UAS promoter when fused to a DBD. These results further confirmed that VIP2 functions as a transcriptional regulator *in planta*, corroborating the findings obtained in the yeast-one hybrid assay (see Fig. 1a).

### VirE2 interacts with the C- terminal NOT domain of VIP2 in yeast expression system

**Figure 2:**
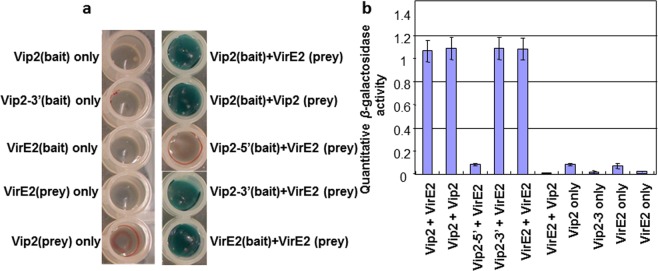
Yeast-two hybrid assay suggests the C-terminal of VIP2 protein interacts with VirE2. These various domains of VIP2 and full length VirE2 were cloned into yeast two-hybrid vectors *pXDGAT*-*CY86* and *pGADT7*, respectively, and were independently transformed into AH109 and MaV204K yeast strains, followed by mating and selection in triple dropout media. (**a**) The LacZ expression in the various bait and bait/prey interactions containing either the full length proteins or the different VIP2 domains. (**b**) Quantitative LacZ activities between various haploid or diploid clones were further confirmed. The experiments were repeated twice with similar results.

VIP2 was identified as a VIRE2 interacting protein using a yeast two hybrid system^13,26^. However, it was not clear which domain of VIP2 interacts with VirE2. It is especially important to know if the NOT domain is important for interaction. To determine this, the *VIP2* gene was split into two regions; a C-terminal fragment containing the NOT2/NOT3/NOT5 plus 445 bp at the 3′ end and an N-terminal fragment minus the NOT domains (5′ end 1440 bp) as described^15^. Based on yeast growth, we concluded that VirE2 interacts with 3′ end of VIP2 that contain the NOT domain (Fig. 2a). In addition, both VIP2 and VirE2 proteins can form a dimer with themselves and activate the reporter genes (Fig. 2a). *LacZ* expression was quantified by β-galactosidase activity to further confirm protein-protein interactions (Fig. 2b).

### Rescuing the tumorigenesis-deficiency phenotype in the *Atvip2* mutant with constitutive expression of the *AtVIP2* gene

**Figure 3:**
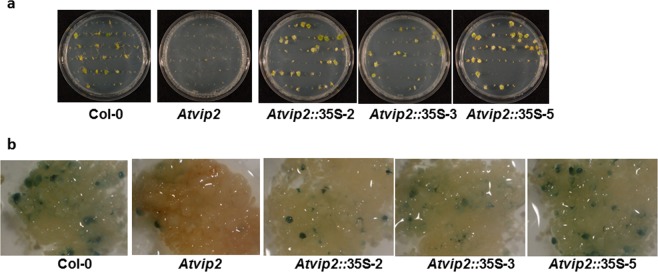
Complementation of the *Atvip2* mutant by constitutive expression of *AtVIP2*. Three independent transgenic events were generated, and molecularly characterized before performing root transformation assays. (**a**) Roots of the wild-type, *vip2* mutant and three transgenic *Atvip2* lines expressing *AtVIP2* cDNA were infected with a tumorigenic strain *A*. *tumefaciens* A208, at 1 × 10^7^ CFU/ml concentration. Tumors incited on the roots were visualized and scored 4 weeks after infection. (**b**) Stable GUS expression. Roots of the wild-type, *Atvip2* mutants and transgenic *Atvip2* lines expressing *AtVIP2* cDNA were inoculated with *A*. *tumefaciens* strain GV3101 carrying the *uidA*-intron gene within the T-DNA at 1 × 10^7^ CFU/ml concentration. The inoculated roots were stained with X-Gluc 2-3 weeks post infection. All the experiments were repeated two times with similar results.

Previously, it was reported that the *Atvip2* mutant is recalcitrant to stable transformation. However, complementation of the mutant was not shown^15^. Therefore, it is not definite if the transformation recalcitrance phenotype is due to the loss of function of *VIP2*. To address this, a construct containing the *AtVIP2* gene (accession # AT5G59710.1) driven by the *CaMV35S* promoter was transformed into the *Atvip2* mutant line. T1 plants of three independent *Atvip2* lines expressing the *AtVIP2* gene (*Atvip2*::35S-2, *Atvip2*::35S-3 and *Atvip2*::35S-5) were tested for restoration of the tumorigenesis-susceptibility phenotype. Root segments of all three transgenic lines along with the wild-type and the *Atvip2* mutant were inoculated with a tumorigenic *A*. *tumefaciens* strain as described^15^. Four weeks after *Agrobacterium* inoculation, number of tumors produced in each plate was scored. As expected, the wild-type Col-0 was susceptible to transformation and *Atvip2* was recalcitrant. The three transgenic *Atvip2* lines expressing the *AtVIP2* gene produced tumors at similar frequencies to that of Col-0 (Fig. 3a), indicating complementation of the tumorigenesis-deficiency phenotype of the *Atvip2* mutant. Furthermore, another stable transformation assay with a non-tumorigenic *A*. *tumefaciens* strain with *uidA* gene within its T-DNA was performed on *Atvip2* lines expressing the *AtVIP2* gene as previously described^27,28^. GUS staining of calli indicated increased expression of the *uidA* gene in the complemented line compared to the *Atvip2* mutant and the *uidA* expression was comparable to that of Col-0 (Fig. 3b). The data from this stable transformation assay are in accordance with the tumorigenesis data and further strengthen our conclusion that expression of the *AtVIP2* gene in the *Atvip2* mutant could restore the transformation-susceptibility phenotype. Taken together, these data suggest that mutation in the *AtVIP2* gene in the *Atvip*2 mutant is responsible for the transformation recalcitrant phenotype.

### *VIP2* is ubiquitously expressed in major plant organs in arabidopsis

**Figure 4:**
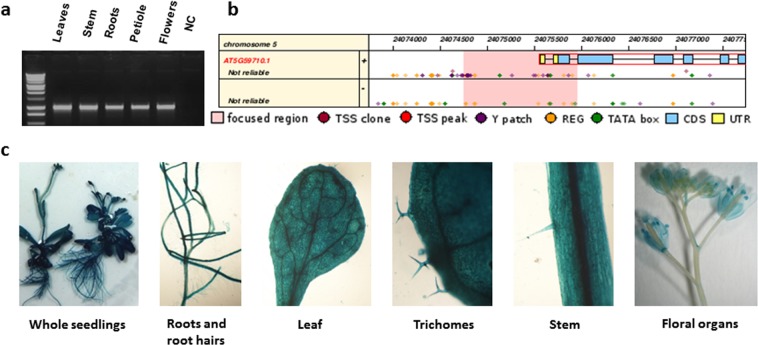
*AtVIP2* gene expression and the *AtVIP2* promoter region. (**a**) The amplification of *AtVIP2* transcripts from different plant organs (leaves, stem, root, petioles and flower) in Arabidopsis by semi-quantitative PCR. NC indicates negative control. (**b**) Schematic presentation of the different regulatory elements on the 1 kb promoter sequence identified upstream of the *AtVIP2* coding sequence as shown in plant promoter database (http://ppdb.agr.gifu-u.ac.jp/ppdb/cgi-bin/index.cgi). (**c**) Analysis of Arabidopsis whole seedlings expressing *GUS* gene under the control of *AtVIP2* promoter. Histochemical GUS staining of transgenic Arabidopsis whole seedlings; roots and root hairs; leaf; trichrome, stem, and floral organs. Pictures were taken 3-4 days after GUS staining.

Expression of the YFP-tagged *AtVIP2* gene in Arabidopsis indicated the cell and tissue-specific expression of the *AtVIP2* gene in the female gametophyte (megasporocytes and tapetum cells) organs, and not in other plant organs^29^. RT-PCR in the same samples showed expression of the *AtVIP2* gene in mature flowers and flower buds, not in leaves, stem and roots^29^. Interestingly, we detected the expression of the *AtVIP2* gene by semi-quantitative RT-PCR in many plant organs including young roots, leaves, stem, petioles and floral tissues (Fig. 4a). These findings contradict previously suggested expression of the *AtVIP2* gene in specific Arabidopsis organelles^29^. To further confirm the *AtVIP2* expression, Arabidopsis transgenic plants expressing an *AtVIP2 Promoter*:*uidA* fusion (Fig. 4b) were developed and 3-4 independent transgenic plants were analyzed by histochemical GUS staining (Fig. 4c). We observed the expression of the *AtVIP2* promoter to be ubiquitous in most plant organs and specifically in the following tissues-: roots, root hairs, leaves, shoots, trichomes, and floral organs (Fig. 4c). Further, based on the GUS staining we concluded that the expression of *AtVIP2* is significantly higher in vasculature and root tissues including lateral roots and root hairs. Additionally, we compared the expression of the *AtVIP2* promoter with the *CaMV35S* promoter. Interestingly, GUS staining in *AtVIP2 Promoter*:*uidA* expressing plants was much stronger in many plant organs when compared to the transgenic Arabidopsis plants expressing a *CaMV35S*-*uidA* construct (Supplementary Fig. S1). Taken together, these data illustrate that the *AtVIP2* gene is ubiquitously expressed in most plant cells and tissues. Our observations are consistent with the information provided at https://apps.araport.org/thalemine/portal.do?externalids=AT5G59710 (Supplementary Fig. S2). More interestingly, we report here the discovery of a plant specific promoter that is potentially stronger and ubiquitous in expression when compared to the *CaMV35S* promoter.

### Transgenic Arabidopsis plants overexpressing the *AtVIP2* gene did not show enhanced transformation

Since the *Atvip2* mutant is recalcitrant to *Agrobacterium*-mediated root transformation, we were interested to determine the effect of overexpressing *AtVIP2* on root transformation. Transgenic Col-0 lines overexpressing *AtVIP2* driven by the *CaMV35S* promoter were generated (Supplementary Fig. S3). A quantitative root tumor assay, as described above, was done using the tumorigenic *A*. *tumefaciens* strain A208. Tumor formation was monitored for a period of four weeks and data were recorded. Our results suggested that overexpression of *AtVIP2* did not significantly improve transformation (Supplementary Figs. S4a, upper panel and S4b, left). The effect of *AtVIP2* overexpression on another stable transformation assay was determined by calculating the frequency of PPT-resistant calli following infection with a disarmed *A*. *tumefaciens* strain GV3101 (*pCAS*1) as previously described^28,30^. There was no significant difference observed in the frequency of PPT-resistant calli formation between *AtVIP2* overexpressing transgenic lines and vector control/or Col-0 wild-type (Supplementary Figs. S4a, lower panel and S4b, right). Taken together, these data suggest that overexpression of *AtVIP2* in *Arabidopsi*s does not improve root transformation.

### VIP2 regulates transcription of defense genes, histones and galactolipid biosynthetic genes

To investigate the role of VIP2 in *Agrobacterium*-plant interaction, we carried out transcriptome profiling by comparing a homozygous Arabidopsis *AtVIP2* overexpressor line with wild-type Col-0 plants following *Agrobacterium* infection. Soil grown Arabidopsis plants in the vegetative stage with fully formed rosette leaves were syringe infiltrated with the disarmed strain of *A*. *tumefaciens*, GV3101 harboring the *uidA*-intron gene within its T-DNA (OD_600_ = 0.2^15^), and samples were collected at 0, 48 and 72 h after infection (HAI). RNA extracted from these samples were analyzed for gene expression using whole genome Affymetrix gene chip (ATH1) microarray. A total of 1,634 genes were differentially expressed (DE) between *AtVIP2* overexpressor line and Col-0. To validate the microarray data, expression of 10 DE genes from *AtVIP2* overexpressor plants were analyzed by RT-qPCR (Supplementary Fig. S5). The results obtained from RT-qPCR are in general agreement with the microarray results.

**Figure 5:**
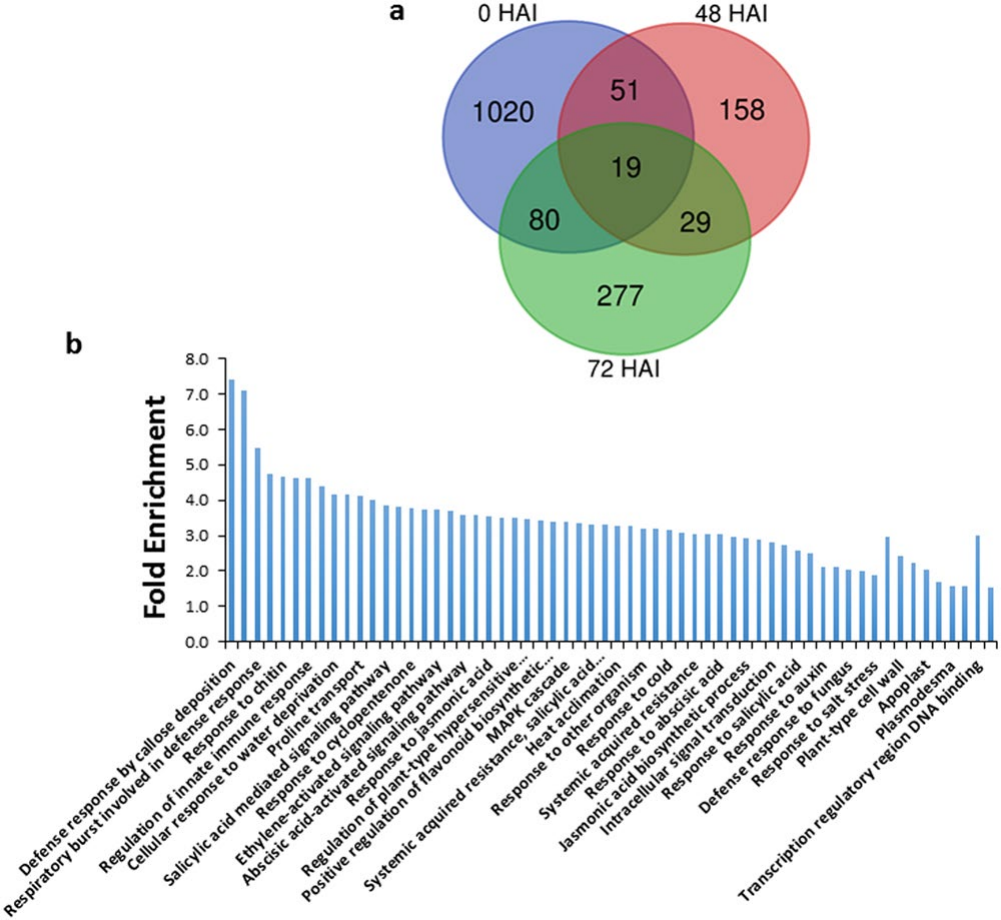
Transcript profiling of *AtVIP2* overexpressor plants during different stages of *Agrobacterium* infection. (**a**) Venn-diagram showing overlap between differentially regulated (DE) genes, compared to Col-0, in *AtVIP2* overexpressor plants during different time points after *Agrobacterium* infection. (**b**) GO term enrichment for up-regulated and down-regulated genes from all time points using Database for Annotation, Visualization and Integrated Discovery.

The Venn diagram for DE genes showed that many DE genes during *Agrobacterium* infection overlapped with DE genes of *AtVIP2* overexpressor plants without *Agrobacterium* infection (Fig. 5a). To study the functional significance of all DE genes, we classified them based on Gene Ontology (GO) term enrichment (Fig. 5b). Various functional categories such as protein targeting to membrane, circadian rhythm, signaling, response to stimulus and regulation of hypersensitive response were enriched. Interestingly, GO terms associated with molecular functions such as sequence-specific DNA binding transcription factor activity and transcription regulatory region DNA binding were enriched. This shows the involvement of VIP2 in transcriptional regulation upon *Agrobacterium*infection with a T-DNA transfer competent strain. Many GO terms associated with stress or immune response were also observed. Interestingly, the majority of the DE genes in *AtVIP2* overexpressor were from 0 HAI i.e., without *Agrobacterium* infection. Surprisingly, many of these genes were also DE in wild-type Col-0 upon *Agrobacterium* infection (Supplementary Fig. S6, Supplementary Table S1). GO term analysis of these commonly DE genes showed that they belonged to various functional categories such as ethylene-mediated signaling, response to ethylene, response to water, cellular response to hormone and defense response. Many genes in these GO terms are known to be involved in plant defense against various stress conditions^31^. Genes regulated by *Agrobacterium* infection and *AtVIP2* overexpression include genes that encode WRKY70, receptor-like protein kinase THESEUS 1, Calmodulin-binding protein 60-like G (CBP60G), Calmodulin like 42 (CML42), Pathogenesis-related protein 5 (PR5), MATE efflux family protein, APS reductase 3, WRKY30 etc. These data suggest that at gene expression level, overexpression of *AtVIP2* in Arabidopsis mimics gene regulation during *Agrobacterium* infection in wild-type Col-0. From the Mapman analysis, we additionally identified many genes involved in proteolysis that were down-regulated at 0 HAI in *AtVIP2* overexpressor plants (Supplementary Fig. S7) compared to Col-0. The above data further strengthens the role for VIP2 as a transcriptional regulator.

An elf-18 inducible gene, *PP2*-*A5* (*phloem protein 2 A5*; At1g65390)^32^, was one the genes showing the highest level of induction in *AtVIP2* overexpressor plants upon *Agrobacterium* infection. Some of the other genes induced are *CAF1A* (*CCR4*-*associated factor 1a*; At3g44260), *β*-*glucosidase* 18 (At1g52400), *QQS* (*Qua*-*Quine Starch*; At3g30720), and *PCC*1 (*Pathogen and Circadian Controlled* 1; At3g22231). Some of the genes with reduced expression during *Agrobacterium* infection in *AtVIP2* overexpressor plants are *TSA1* (*TSK*-*associating protein 1*; At3g15950), *LEA*25 (*Late*-*embryogenesis abundant protein* 25; At2g42560), *LEA4*-1 (At1g32560), *CML*41 (*Calmodulin*-*like protein* 41; At3g50770), and *DEFL* (*Defensin*-*like*; At3g05730).

Genes that were specifically regulated in *AtVIP2* overexpressor plants during *Agrobacterium* infection process (*AtVIP2* overexpressing plants at 48 and 72 HAI compared with *AtVIP2* overexpressing plants at 0 HAI) belonged to GO terms such as galactolipid biosynthetic process, cellular response to phosphate starvation, response to fructose, response to sucrose and UDP-glycosyltransferase (UGT) activity (Supplementary Table S2). These GO categories were observed from the down-regulated data set. UGTs transfer glycosyl residues from activated nucleotide sugars to acceptor molecules (aglycones) containing an aromatic ring^33^. They were found to be involved in plant disease and defense responses. We also found that various histone genes that have been shown to be down-regulated in an *Atvip2* mutant^15^ and a *not2a Atvip2* mutant^21^, are up-regulated in our data set (Supplementary Fig. S8). Some up-regulated genes identified in our study encode proteins such as Arabinogalactan, CAF1, Nodulin-Like Protein and Protein Phosphatase 2 C which have been previously reported to be important for *Agrobacterium*-mediated plant transformation^34–37^.

### ABRE motif containing genes are repressed in *AtVIP2* overexpressors

*Cis*-acting elements are key regulators of gene expression. Therefore, *in silico* motif analysis to identify motifs that are specifically enriched in the promoters of DE genes was performed. Enrichment of mCACGTGk motif in the down-regulated gene set was identified (Supplementary Tables S3, S4). ACGTG is a core of the Abscisic Acid Responsive Element (ABRE) that is involved in the abscisic acid (ABA)-regulated gene expression^38^, and genes containing them responds to abiotic stress including drought stress and ABA treatment^39^. ABA, an abiotic stress hormone, has a major role in regulation of physiological processes during abiotic stress responses^40^. Some reports suggest that ABA is also involved in plant defense signaling against pathogens and is an essential component in integrating and fine-tuning abiotic and biotic stress-response signaling networks^41,42^. The above reports are in support of the GO term analysis, in that many of the signaling pathways enriched in our study (Fig. 5) have cross-talk with each other (reviewed in^43^). On the other hand, we did not find significantly enriched motifs in up-regulated gene set.

### *AtVIP2* overexpression causes low ABA content and sensitivity to abiotic stresses

**Figure 6:**
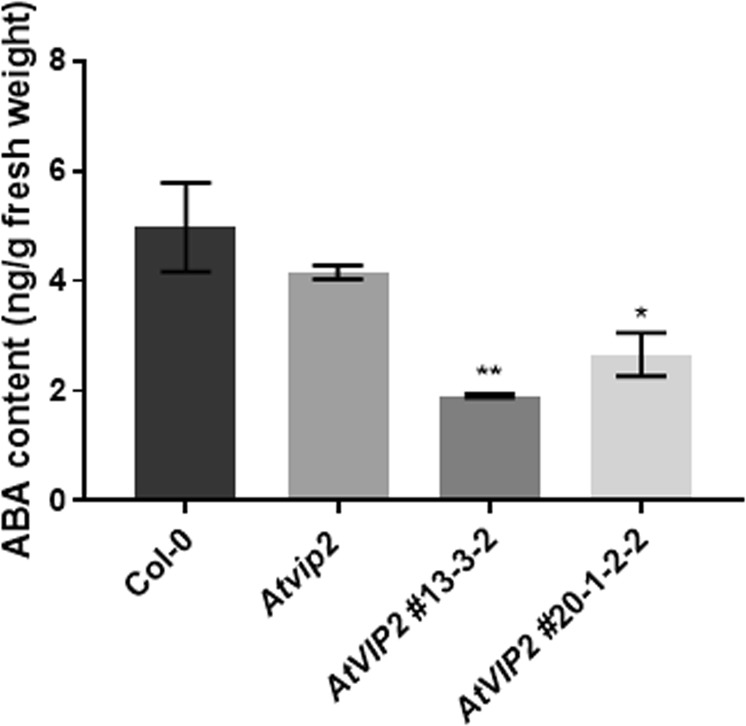
ABA content of wild-type, *Atvip2* mutant and *AtVIP2* overexpression lines. Three weeks old seedlings grown in half MS media were used for ABA quantification. Error bars indicate SE of the mean (n ≥ 8). Asterisks indicate significant differences (*P ≤ 0.05 and **P ≤ 0.005) between Col-0 and other plants, as determined by two-way ANOVA, uncorrected Fisher’s LSD test.

**Figure 7:**
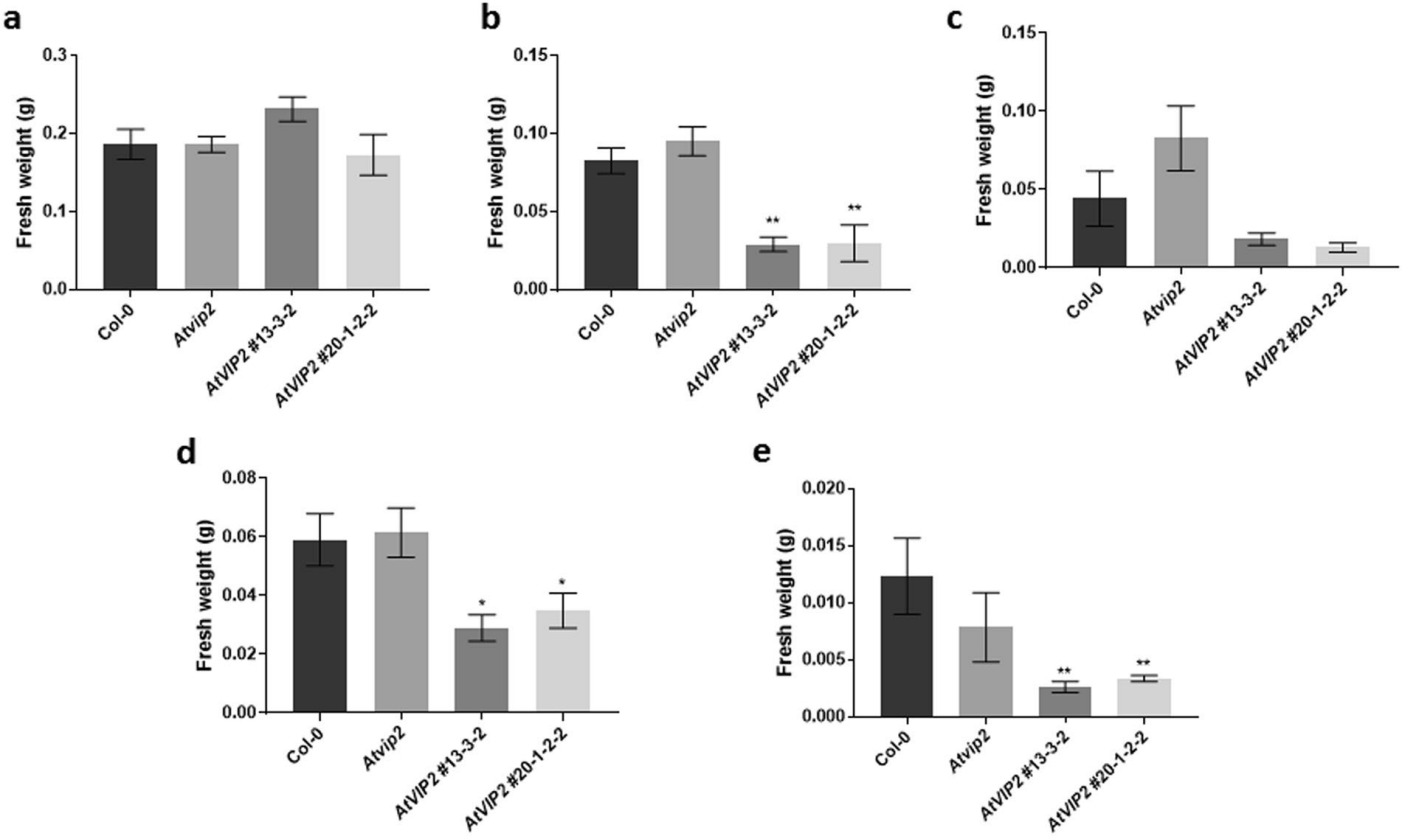
Effect of abiotic stress conditions on the growth of wild-type, *Atvip2* and *AtVIP2* overexpressor plants. Growth of plants under (**a**) normal (half strength MS), (**b**) 5 µM ABA, (**c**) 10 µM ABA, (**d**) 75 mM mannitol and (**e**) 100 mM NaCl. In all graphs, error bars indicate SE of the mean (n ≥ 8). Asterisks indicate significant differences (*P ≤ 0.05 and **P ≤ 0.005) between Col-0 and other plants, as determined by two-way ANOVA, uncorrected Fisher’s LSD test.

Since we observed enrichment of ABRE motifs in the promoters of down-regulated genes of *AtVIP2* overexpressor plants, we further determined the ABA levels and the response of the *AtVIP2* overexpressor lines to abiotic stress. We quantified the level of ABA in wild-type Col-0, *AtVIP2* overexpressor, and *Atvip2* knockout plants grown on half strength MS for three weeks. *AtVIP2* overexpressor lines showed significantly less ABA content than Col-0 plants (Fig. 6). In addition, we studied response of *Atvip2* and *AtVIP2* overexpressor plants to various abiotic stresses by transplanting seedlings to half strength MS plates containing 100 mM NaCl, 5 µM ABA, 10 µM ABA or 75 mM mannitol. *AtVIP2* overexpressor plants had lower fresh weights compared to wild-type plants when grown in 5 µM ABA, 100 mM NaCl or 75 mM mannitol (Fig. 7). The above data suggest that AtVIP2 regulates abiotic stress responses in plants, which is consistent with the speculated role as transcription regulator.

## Discussion

*Agrobacterium*-mediated plant transformation is a complex process involving the functions of both bacterial virulence proteins and host proteins in various steps of the transformation process. Roles for plant genes in T-DNA transfer and integration during the transformation process have been shown or proposed^44–46^. *VIP2* is one such plant gene required for *Agrobacterium*-mediated stable plant transformation^15^ and acts as a general transcriptional regulator controlling plant development^21^. In the present study, surprisingly, we showed that overexpression of *AtVIP2* in Arabidopsis did not have a significant impact on *Agrobacterium*-mediated plant transformation. Our finding contradicts the data from Zhao and coworkers^47^ that illustrates up to 2.5-fold increase in *Agrobacterium*-mediated transformation efficiency in tobacco by the heterologous expression of the *Triticum aestivum VIP2*. It is not clear if endogenous *VIP2* is expressed at saturated levels in tobacco. Nevertheless, these results show that over-expression of *VIP2* can enhance transformation in some plant species, further confirming the role of VIP2 in *Agrobacterium*-mediated plant transformation. Another possible reason for the differences observed between our study, and Zhao *et al*.^47^ may be the use of homologous versus heterologous *VIP2* gene sequences for overexpression. In a previous study, it has been shown that overexpression of *AtVIP1* (a bZIP protein) in Arabidopsis did not alter either transient or stable transformation susceptibility^48^, whereas T-DNA transformation efficiency was improved when *AtVIP1* expressing transgenic tobacco plants were retransformed with *Agrobacterium*^49^. Though the increased transformation efficiency was attributed to the lack of use of full-length *VIP1* cDNA^48,49^, it is interesting to note that homologous expression of *VIP1* did not alter transformation efficiency in Arabidopsis similar to our study. Based on the above findings, we speculate VIP2 is necessary but not a rate-limiting protein in *Agrobacterium*-mediated transformation in Arabidopsis.

A previous study^22^ showed that VirD5 interacts with AtVIP2 and competes with it for binding with cap binding proteins (CBPs; CBP20 and CBP80) that negatively regulates the *Agrobacterium* infection process. The interaction between AtVIP2 and VirD5 is very specific in that VirD5 interacts with AtVIP2 of Arabidopsis, but not with the homolog NOT2a of Arabidopsis or the ortholog OsNOT2 of rice^22^. It would be interesting to understand the role of AtVIP2 and VirD5 interaction in *Agrobacterium*-mediated plant transformation. In our previous publication, we demonstrated that VIP2 interacts with VIRE2^15^. Wang *et al*.^22^ also showed that VIP2 and VIRE2 along with VIRD5 form a ternary complex *in vivo*. In this study, we specifically showed that VIRE2 interacts with C-terminal NOT domain of VIP2. We speculate that VIRE2 interacts with VIP2 to alter VIP2’s activity, which in turn will regulate expression of genes such as histones to enhance *Agrobacterium*-mediated plant transformation.

In this study, we also investigated *AtVIP2* promoter activity in multiple tissues or organs and showed that the *AtVIP2* promoter is highly active and expressed constitutively. *Agrobacterium* infection was previously shown to induce *AtVIP2* expression in Arabidopsis^15^. We speculate that the VIP2 protein is present at saturated levels in Arabidopsis and therefore overproduction of this protein does not have an impact on plant transformation. However, overexpression of *AtVIP2* resulted in differential expression of many genes that are important for *Agrobacterium*-mediated plant transformation (Fig. 5) including up-regulation of histone genes (Supplementary Fig. S8). In addition, expression of defense related genes were modulated in the *AtVIP2* overexpressor plants. During *Agrobacterium* infection process, plant genes necessary for the transformation process are induced and host defense genes are repressed^50,51^. Surprisingly, we also found many genes involved in proteolysis were down-regulated in *AtVIP2* overexpressor plants (Supplementary Fig. S7). Proteolysis of proteins coating the T-DNA is important for subsequent integration of T-DNA to the host genome^52^. It would be interesting to study how VIP2 affects proteolysis.

Genome-wide transcript profiling of *Agrobacterium*infected *AtVIP2* overexpressor lines revealed the presence of many differentially regulated genes and GO terms. UGT is one of the GO terms specifically enriched in *AtVIP2* overexpressor plants during *Agrobacterium*infection (Supplementary Table S2). Overexpression of barley *UGT* in wheat resulted in enhanced resistance to *Fusarium graminearum*^53^. Recently, it has been shown that expression of wheat *UGT* in tobacco and Arabidopsis reduced the efficiency of *Agrobacterium*-mediated plant transformation^54^. Though we did not find any relevant literature related to the galactolipid biosynthetic process or cellular response to phosphate starvation in relation to *Agrobacterium*-mediated plant transformation, it is important to note that alteration of membrane lipid composition is one of the adaptive mechanisms in higher plants to cope with phosphate starvation^55^. Highly induced genes in *AtVIP2* overexpressor plants upon *Agrobacterium* infection include *CAF1A*, *β*-*glucosidase* 18, *QQS* and *PCC1*. *CAF1A* is involved in mRNA deadenylation and mediation of stress responses^56^. β-glucosidase 18 plays a role in the metabolism of abiotic stress hormone ABA^57^. An orphan gene, *QQS*, functions in modulation of carbon and nitrogen allocation^58^. RNAi silencing of *PCC1*, a regulator of defense against pathogens and stress-activated transition to flowering, resulted in plants being more susceptible to hemi-biotrophic oomycete pathogen, *Phytophthora brassicae*, and more resistant to the necrotrophic fungal pathogen *Botrytis cinerea*^59^.

GO terms related to abiotic stress responses such as response to salt stress, response to ABA, etc. are enriched among DE genes (Fig. 5). Motif analysis of DE genes dataset resulted in identification of ABRE containing motif (mCACGTGk) in the promoters of the down-regulated genes (Supplementary Table S3), and enrichment of these motifs in the 72 HAI down-regulated DE genes dataset (Supplementary Table S4). At 72 HAI, many abiotic stress responsive genes such as late embryogenesis abundant proteins (LEA) (At5g06760, At3g15670, At1g52690 and At2g35300), and calmodulin-like protein 41 (CML41) (At3g50770) were down-regulated. Endogenous ABA levels are an important factor in shaping plants tolerance to various abiotic stresses^60–62^. ABA induces expression of ABA-responsive genes via ABREs in their promoter regions^60,63^. ABRE containing elements are enriched in promoters of genes that are responsive to various abiotic stresses^64^. LEA proteins are intrinsically disordered proteins, have major role in abiotic stress tolerance in plants^65,66^. CMLs are major Ca^2+^ sensors, and they target many genes involved in various developmental processes and stress tolerance^67,68^. *CML41* was induced by both plant cell wall derived oligosaccharides and bacterial flagellin peptide Flg22, and have been suggested to have a role in dampening plant immune responses^69^. In addition, recently, *CML41* has been shown to have increased expression in response to elevated temperature^70^. Expression of *TSA1* and *DEFL* was also reduced in *AtVIP2* overexpressor plants upon *Agrobacterium* infection. *TSA1* is involved in MeJA-induced ER body formation in plants^71^. Similar to our study, infection of a fungal pathogen *Alternaria brassicicola* represses the expression of *DEFL*^72^.

Results from our study along with previous reports^15,21^ showed that VIP2 is not only a regulator of plant genes involved in *Agrobacterium*-mediated plant transformation process but also a general transcriptional regulator that control multiple pathways. Our results showed that overexpression of *AtVIP2* in Arabidopsis resulted in differential regulation of many abiotic stress related genes. This observation is consistent with the observation that the NOT2 domain of *Fusarium oxysporum* is suggested to regulate vegetative growth, conidiogenesis and virulence of the fungus by the transcriptional regulation of genes involved in multiple pathways controlling cell wall integrity, oxidative stress response, ROS production and fusaric acid production^73^ suggesting that VIP2 controls several physiological and metabolic pathways.

Modulating *VIP2* expression might be a useful tool for enhancing plant transformation efficiency in plant species where endogenous *VIP2* expression is at low level. However, susceptibility of *VIP2* overexpressor lines to ABA and other abiotic stresses (Fig. 7) can be an issue. It requires further studies to understand how VIP2 regulates abiotic stress responses.

## Methods

### Yeast one-hybrid assay

The yeast one-hybrid assay was performed as described earlier^24^. VIP2 (GenBank # AF295433) open reading frame was fused to the GAL4 DNA binding domain (BD) and cloned into a *pGBKT7* yeast vector containing the auxotrophic marker *trp1* gene and the *GAL4* upstream activating sequence (UAS promoter). The human *lamin C* and *topoisomerase I* genes cloned into the *pGBKT7* vector were used as controls. These two genes are known to function as non-specific activators of the auxotrophic marker, but can be deactivated by the addition of 3-amino-1,2,4-triazole (3-AT). All clones were transformed into the MaV204K yeast strain. A single colony of each transformed MaV204K was picked and grown overnight in 100 µl liquid synthetic dropout (SD) medium lacking tryptophan in 96 well plates. All cultures were transferred into a new 96 well plate with three different dilutions of the liquid culture (1:10, 1:100 and 1:1000). Cultures were plated onto solid SD medium, lacking tryptophan and containing 3-AT, using 96-well pin replicator, and incubated at 30 °C overnight for the single-dropout assay. The three clones fused to the GAL4-BD were also replicated on SD medium lacking tryptophan and histidine, and containing 10 mM 3-AT for the double-dropout assay.

### Transactivation analysis

A promoter containing three copies of the GAL4 upstream activating sequence (UAS), and VIP2 were fused to the DBD of GAL4 modified for optimal expression in plants (mGAL4) and expressed constitutively under the *CaMV*35*S* promoter from a *pSAT6* vector (*pSAT6*-*mGAL4*-*DBD*-*VIP2*). Additionally, the *pSAT6*-*uasP*-*GUS* vector^25^ with *GUS* reporter gene fused to the minimal *UAS* promoter (*uasP*) was also included as a control. The biolistic approach for DNA delivery was performed using standard protocols and plasmids as described^25^.

### Bait and Prey Construction

The full-length, C-terminal, and N-terminal of *VIP2* and the *VirE2* genes were PCR amplified using a high-fidelity platinum *Pfx* DNA polymerase (Invitrogen Inc.) and cloned into the pENTR/D Topo vector (Invitrogen, Carlsbad, CA). All these clones were then cloned into the yeast two-hybrid DNA BD vector *pXDGAT*-*CY86* with the cyclohexamide sensitive gene (*CYH*^*S*^)^74^. The full-length *VIP2* and *VirE2* genes were also cloned into the DNA activation domain (AD) domain vector, *pGADT7*-*Rec7G* to be used as preys. The prey vector *pGADT7*-*Rec7G* was constructed from the *pGADT7*-*Rec7* vector (Clontech, Mountain View, CA) by introducing the GATEWAY cassette at the *Sma*I site using standard cloning protocols and confirmed by sequencing. Recombination between *pENTR/D* Topo vectors and the destination vector was performed using the Clonase II enzyme mix according to the manufacturer’s instructions (Invitrogen, Carlsbad, CA). Bait and prey constructs were confirmed to be in-frame with the DNA-BD or DNA-AD prior to transformations. Bait constructs were transformed into the MaV204K yeast strain (*MAT* α, *leu2*-3,112; *trp1*-901; *his3* Δ200; *ade2*-101; *cyh2*^*R*^; *can1*^*R*^; *gal4 Δ*; *gal80 Δ*; *GAL1*::*lacZ*;*HIS3UASGAL1*::*HIS3@LYS2*; *SPAL10*::*URA3*)^75^. Prey constructs were transformed into AH109 yeast strain (*MATa*, *trp1*-901, *leu2*-3, 112, *ura3*-52, *his3*-200, *gal4* Δ, *gal80* Δ, *LYS2::GAL1UAS*-*GAL1TATA*-*HIS3*, *GAL2UAS*-*GAL2TATA*-*ADE2*, *URA3::MEL1UAS*-*MEL1TATA*-*lacZ*, *MEL1*). All transformations were performed as described^76^. All bait strains were checked for auto activation of the reporter gene, *his*, by checking their growth on SD -His/-Trp supplemented with 0, 2.5, 5, 7.5, 10 and 15 mM 3-AT. The 3-AT concentration of 10 mM was found to inhibit auto activation of the both bait or prey clones, so it was used as media supplement in the rest of the study.

### Mapping the interaction between VirE2 with VIP2

Pairs of various bait and prey constructs were co-transformed into AH109 strain to test the specificity and to map the interaction between VirE2 and VIP2 (Fig. 2a). Co-transformants were selected on solid SD medium lacking Ade, His, Trp, and Leu and supplemented with 10 mM 3-AT and X-gal. Positive clones were then re-grown in SD liquid medium under the same selection conditions. The selection was repeated four times by sub culturing positive clones and assayed for the activity of X-gal. Clones that maintained an ability to grow under selected conditions were then grown on liquid SD medium in a 96-well (Fig. 2a, left panel) plate along with yeast strains containing only bait or prey constructs as negative controls (Fig. 2a, right panel).

### Validation and β-galactosidase assays

Co-transformed yeast strains with various *VIP2* and *VirE2* constructs, and only bait or prey, were grown on 96 well plates containing liquid SD medium that lacks Ade, His, Trp, and Leu supplemented with 10 mM 3-AT for 24 h. Absorbance at A_600_ was measured for three independent 100 µl cultures and then assayed for β-galactosidase activity using the yeast β-galactosidase assay kit (cat. 75768; Pierce Biotechnology, Inc.) following the manufacturer’s instructions. A_600_ and A_420_ were measured using multi-plate reader, Tecan infinite 200Pro. The β-galactosidase activity was calculated using the equation: β-galactosidase activity = 1000 × A_420_/T × V × A_600_ where T is time (in minutes) of incubation and V is volume of cells (ml) used in the assay.

### Plant transformation experiments

We tested whether constitutive overexpression of *AtVIP2* in the *Atvip2* mutant and Col-0 plants could restore the wild-type phenotype and/or increase the transformation efficiency. Briefly, for over-expression studies, cDNA corresponding to the Arabidopsis *VIP2* cDNA was amplified by RT-PCR along with GATEWAY adapter primers, sequence verified and cloned into the plant expression vector *pMDC32*^77^ driven by constitutive *CaMV*35*S* promoter. Wild-type Arabidopsis Col-0 and *Atvip2* mutant plants were transformed by the floral dip method^78^. The transgenic plants were selected for resistance to hygromycin and also tested for the presence of the transgene using the GATEWAY adapter primers (attB1/B2), while the expression of the transgene was confirmed by semi-quantitative RT-PCR using gene specific primers. These transgenic plants were selfed and T1 homozygous seeds were collected.

### Root tumor, GUS and callus assays

Arabidopsis root tumor assays were performed as described earlier^15,28,30^. For root tumor assays, axenic root segments from wild-type Col-0 and transgenic plants were infected with a tumor-inducing *A*. *tumefaciens* strain, native A208 containing the nopaline type Ti-plasmid *pTiT3*, co-cultivated for 48 h in the dark at room temperature and transferred to a hormone-free Murashige and Skoog media (MS) supplemented with cefotaxime (250 mg/l) and timentin (100 mg/l). Tumor numbers and phenotypes were recorded at 4 weeks after infection. Stable GUS transformation assays were performed as described earlier^27,39^. Root segments from Col-0 and transgenic plants were infected with disarmed strain of *A*. *tumefaciens* GV3101 carrying *uidA*-intron gene within the T-DNA and co-cultivated for 48 h at room temperature. The root segments were incubated on callus induction medium (CIM) for 2-3 weeks, and stained with X-gluc. Stable transformation callus assays were carried out as described earlier^28,30^. Root segments from wild-type Col-0 and transgenic plants were infected with a disarmed *A*. *tumefaciens* strain GV3101 containing *pCAS1*^28^ and co-cultivated for 48 h in dark at room temperature. The root segments were incubated on CIM supplemented with phosphinothricin (PPT) at 10 mg/l, cefotaxime (250 mg/l) and timentin (100 mg/l). The number of root segments forming PPT-resistant calli was counted at 4 weeks after infection.

### Tissue specific expression of *VIP2*

The promoter sequence upstream of the ORF (1 kb region, see ppdb: Plant promoter database, http://ppdb.agr.gifu-u.ac.jp/ppdb/cgi-bin/index.cgi, Fig. 4b)^79^ was PCR amplified, confirmed by sequencing, and fused to a reporter gene (*uidA*) in the *pMDC162* vector, and mobilized into *A*. *tumefaciens* strain GV3101. Arabidopsis plants were transformed with the *VIP2 Promoter*:*uidA* fusion expression cassette via the floral dip transformation method and hygromycin-resistance transgenic T1 events were identified for promoter expression analysis. Three to four independent T1 lines were selected and screened by histochemical staining for GUS expression.

### Microarray gene expression analysis

Three biological replicates were performed for each tissue sample. Total RNA was isolated as described previously^15^. RNA was quantified and evaluated for purity using a Nanodrop Spectrophotometer ND-100 (NanoDrop Technologies, Willington, DE) and Bioanalyzer 2100 (Agilent, Santa Clara, CA). Ten μg of total RNA was used for the expression analysis of each sample using the Arabidopsis ATH1 chip (Affymetrix, Santa Clara, CA). Probe labeling, chip hybridization and scanning were performed per the manufacturer’s instructions for one-cycle labeling (Affymetrix). Data normalization between chips was conducted using RMA (Robust Multichip Average)^80^. Presence/absence calls for each probe set were obtained using dCHIP^81^. Gene selections based on Associative T-test^82^ were made using Matlab (MathWorks, Natick, MA). In this method, the background noise present between replicates and technical noise during microarray experiments was measured by the residual present among a group of genes whose residuals are homoscedastic. Genes whose residuals between the compared sample pairs that are significantly higher than the measured background noise level were considered to be differentially expressed. A selection threshold of 2 for transcript ratios and a Bonferroni-corrected *P* value threshold of 2.19202E-06 were used. The Bonferroni-corrected *P* value threshold was derived from 0.05/N in these analyses, where N is the number of probes sets (22,810) on the chip.

Functional classification of genes was carried out using the Database for Annotation, Visualization and Integrated Discovery (DAVID) version 6.7^83^ with the Benjamini correction and false discovery rate (FDR) <0.05, as well as MAPMAN (http://mapman.gabipd.org/) using log_2_ transformed value of transcript ratios.

### *In silico* motif analysis

The 500 bp upstream region of one hundred genes from each class was downloaded from RSAT site (http://floresta.eead.csic.es/rsat/) with the option preventing overlap with neighbor genes (noorf). Promoters were analyzed using the online tool MotifSampler (http://bioinformatics.intec.ugent.be/MotifSuite/motifsampler.php). A precompiled background model of Arabidopsis with order set to 3 was used. The total number of runs was set to 50. MotifRanking (http://bioinformatics.intec.ugent.be/MotifSuite/motifranking.php) was used to rank highest scoring motifs based on LogLikelihood ratios.

### ABA quantification

Freshly harvested plant tissues were frozen in liquid nitrogen and ground to a fine powder, and stored at −80 °C until ABA extraction. ABA quantification was carried out as described^84^. In brief, 1 ml of cold methanol:water (70:30, v-v) plus labeled ABA was added to 100 mg of powdered tissue. Samples were vortexed, sonicated, and extracted at 4 °C for 1 h. Samples were centrifuged at 16,000 × g for 5 min at 4 °C and the supernatants were dried using nitrogen gas. The residues were re-dissolved in 100% methanol, and the supernatant injected into an Agilent 1290 UHPLC connected to an Agilent 6430 Triple Quad mass spectrometer (Agilent Technologies). Separation was carried out using a Waters BEH C18 column (Waters Co., 1.76 µm, 2.1 × 150 mm). The following solvents were used at a flow rate of 0.4 ml min^−1^: (A) 0.05% formic acid/H_2_O and (B) acetonitrile/0.05% formic acid. The separation was achieved by starting with 5% solvent B, a gradient from 5% to 46% of solvent B over 19 min and a step to 90% B in 0.1 min, then a hold at 90% B for 2 min and a step to 5% solvent B in 0.1 min. The temperature of the UPLC column was set to 40 °C. The gas temperature was 300 °C, gas flow: 9 ml/min, nebulizer was 25 psi. Fragmentor and collision energy were optimized for each compound individually. The SRM analysis conditions for ABA and d_6_ABA (negative ion mode) were as follows: capillary = 4,000 V, fragmentor voltage = 100 V, collision energy = 4 V, dwell time = 200 ms and SRM transition (*m/z*) = 263/153 for unlabeled ABA and 269/159 for d_6_ABA. Relative amounts of ABA were based on comparison to the labeled hormone.

### Abiotic stress experiments

Seeds of Arabidopsis ecotype Col-0 were surface sterilized in 75% ethanol for 2 min, and 30% bleach for 15 min, followed by four washes with sterile water. The seeds were sown onto half-strength MS medium containing 1% sucrose and 0.3% phytagel, stratified in the dark at 4 °C for 2 d and grown in growth chamber with 12.5 h day length at 24 °C. Seven days after stratification, seedlings were transferred to half-strength MS plates containing 5 µM ABA, 10 µM ABA, 75 mM mannitol, and 100 mM NaCl. Fifteen days after transfer, fresh weights of the plants were measured.

## Supplementary information

Supplementary Data

## Data Availability

All the data presented in the manuscript is publicly available. All transcriptome data is loaded into ArrayExpress with accession # E-MTAB-8326.
