# Supplementary material for: Overexpression of VIRE2-INTERACTING PROTEIN2 in Arabidopsis regulates genes involved in Agrobacterium-mediated plant transformation and abiotic stresses
Source: Sci Rep. 2019 Sep 18;9:13503. doi: 10.1038/s41598-019-49590-3 (PMC6751215; doi:10.1038/s41598-019-49590-3)
Supplement: Supplementary file 1 — Supplementary Data [file 41598_2019_49590_MOESM1_ESM.pdf]

Overexpression of *VIRE2-INTERACTING PROTEIN2* in Arabidopsis regulates genes involved in *Agrobacterium*-mediated plant transformation and abiotic stresses

Vidhyavathi Raman, Ajith Anand, Balaji Vasudevan, Mustafa R. Morsy, Bikram D. Pant, Hee-Kyung Lee, Yuhong Tang and Kirankumar S. Mysore

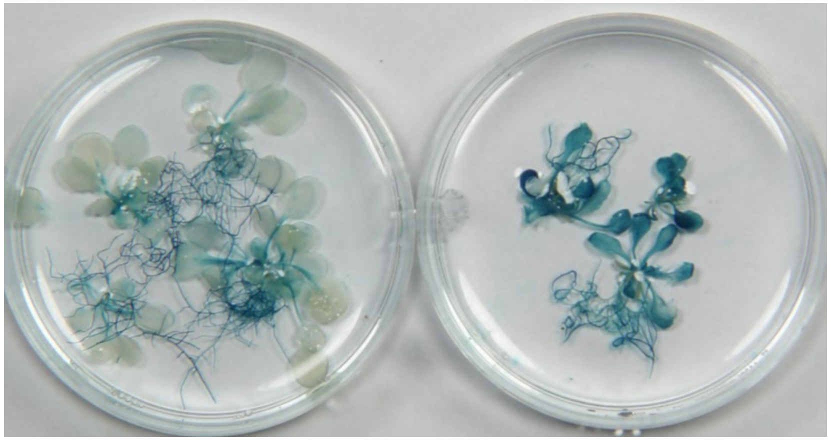

Supplementary Figure S1. Analysis of Arabidopsis whole seedlings expressing *GUS* gene under the control of *CaMV 35S* and *AtVIP2* promoters. Histochemical staining of multiple events showed higher *GUS* expression in the events with *AtVIP2* promoter as compared to *CaMV 35S* promoter.

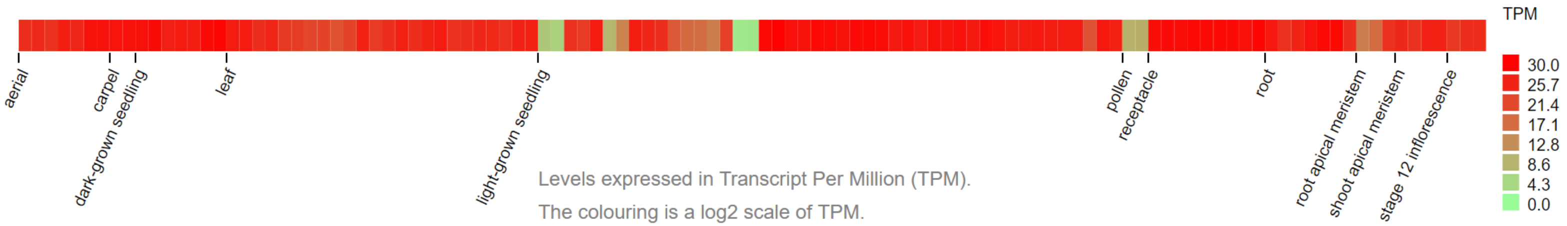

Supplementary Figure S2. ThaleMine expression map describing the *AtVIP2* expression pattern over Arabidopsis development.

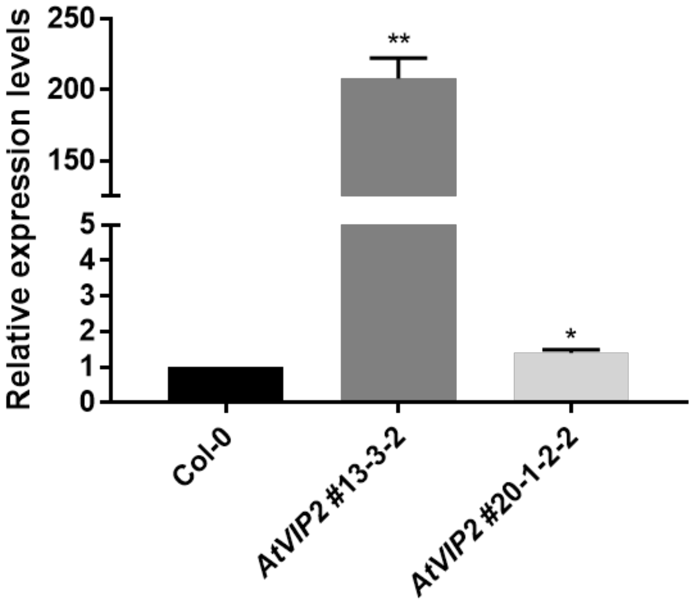

Supplementary Figure S3. Expression of *AtVIP2* gene in wild-type, mutant and overexpression lines. Three weeks old seedlings grown in half strength MS were used for RT-qPCR. Expression levels were calculated using $2^{-\Delta\Delta CT}$ method with *Actin* gene as a house keeping control. Error bars indicate the SE of the mean (n ≥ 8). Asterisks indicate significant differences (*P ≤ 0.05 and **P ≤ 0.005) between Col-0 and overexpressor plants, as determined by Student's *t* test.

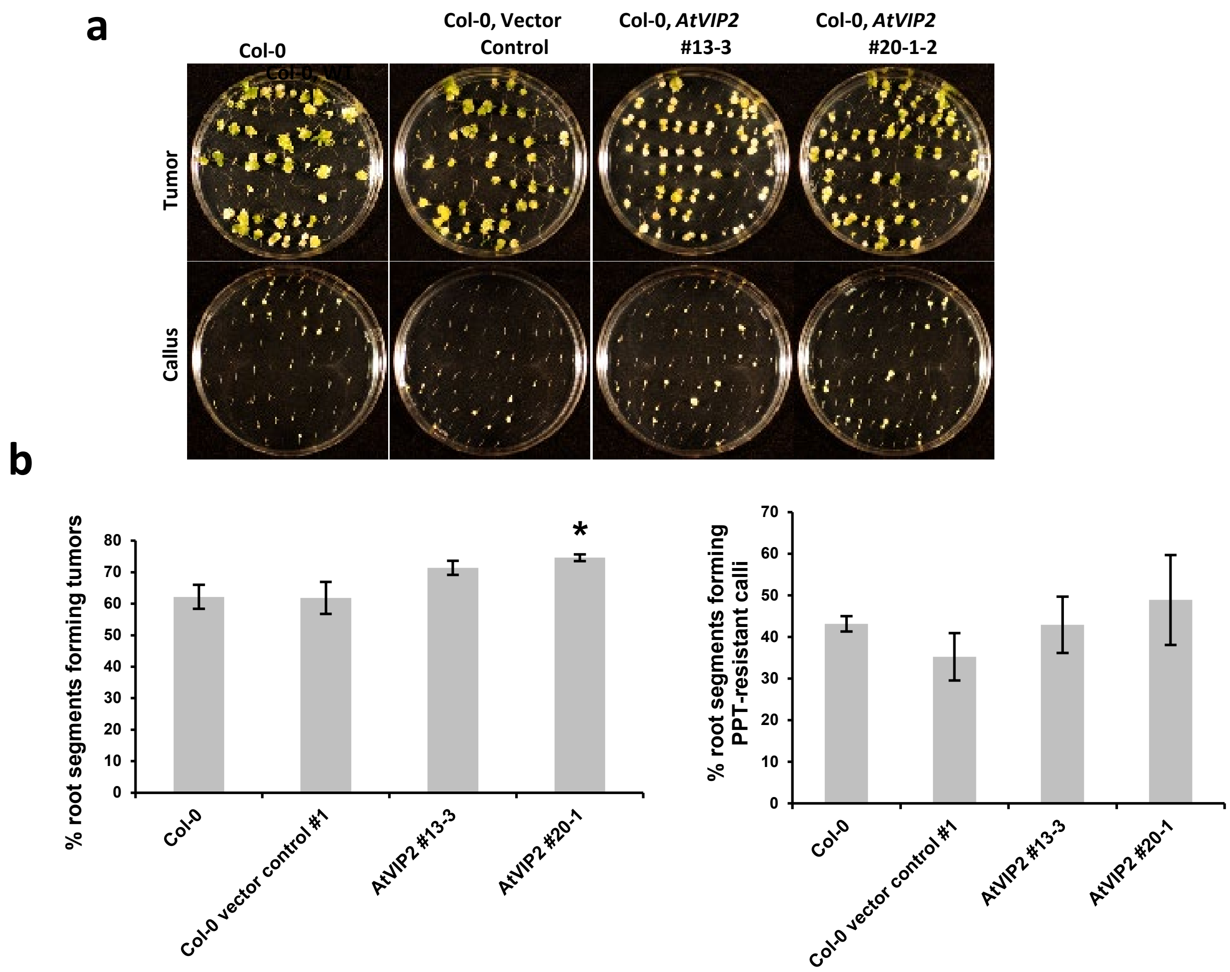

Supplementary Figure S4. Root tumor and callus assay to determine the effect of *AtVIP2* overexpression on stable transformation efficiency in *Arabidopsis*

(**a**) Representative plates of tumor and callus assay from Col-0 versus *AtVIP2* overexpressing transgenic lines. Root segments of Col-0 and transgenic lines over-expressing *AtVIP2* were infected with tumorigenic strain A208 at 1 x 10^6 CFU/ml concentration. Tumors developing from root segments were visualized and scored four weeks after infection (upper panel). Root segments from all the above mentioned lines were also infected with disarmed *A. tumefaciens* strain GV3101 harboring the binary vector *pCAS1* (that contains *bar* gene as plant selection marker) at 1 x 10^6 CFU/ml concentration. Phosphinothricin-resistant calli developing from root segments were visualized and scored four weeks after infection (lower panel). (**b**) Bar graph showing the frequency of tumor (left) and calli (right) formation in infected root segments. Data is presented as mean ± SD (n = 3) from three replicates. Asterisks indicate a significant difference in frequency of tumor (left panel) between Col-0/Col-0 vector control and one of the transgenic line expressing *AtVIP2* according to Student's *t* test (P <0.05).

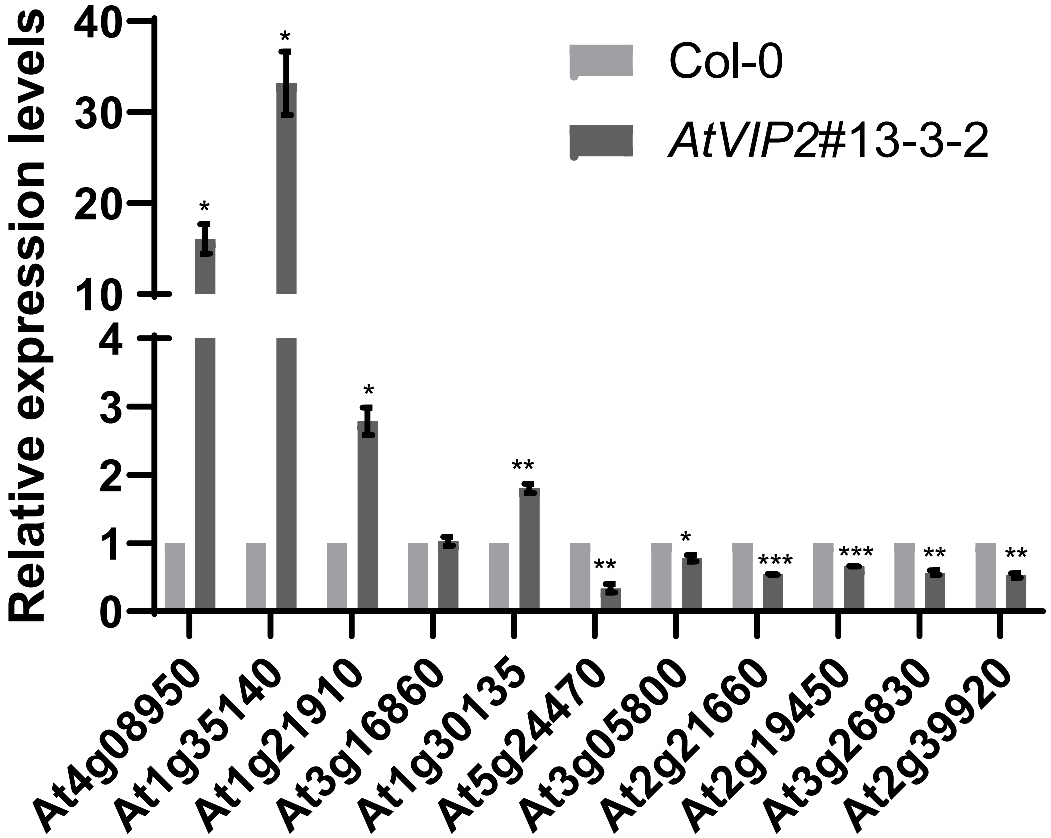

Supplementary Figure S5. Validation of the microarray data by real-time RT-qPCR. Differential expression of 10 genes that had more transcript abundance or less transcript abundance in *AtVIP2* overexpressor plants compared with Col-0 in the microarray experiment were selected for validation by real-time RT-qPCR. Three weeks old seedlings grown in half strength MS were used for RT-qPCR. Expression levels were calculated using $2^{-\Delta\Delta CT}$ method with *UBQ10* gene as a house keeping control. Error bars indicate the SE of the mean (n ≥ 8). Asterisks indicate significant differences (*P ≤ 0.05, **P ≤ 0.005 and ***P ≤ 0.0005) between Col-0 and overexpressor plants, as determined by one sample *t* and Wilcoxon test.

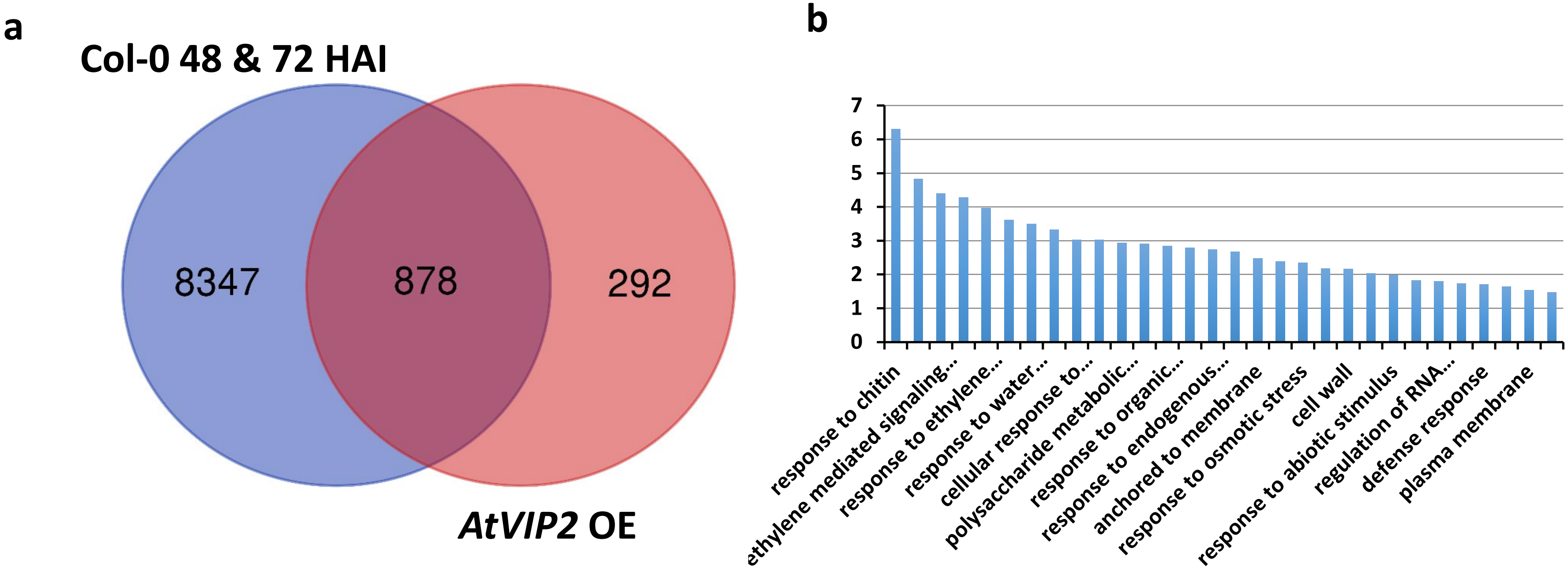

Supplementary Figure S6. Many of the DE genes in *AtVIP2* overexpressor (OE) lines (0 HAI) are induced by *Agrobacterium* in Col-0. (**a**). Venn-diagram showing overlap between differentially regulated (DE) genes in *AtVIP2* OE plants at 0 HAI and *Agrobacterium* infected Col-0 plants (both 48 and 72 HAI). (**b**). GO term enrichment for 878 DE genes that are common between above two sets of genes, using DAVID.

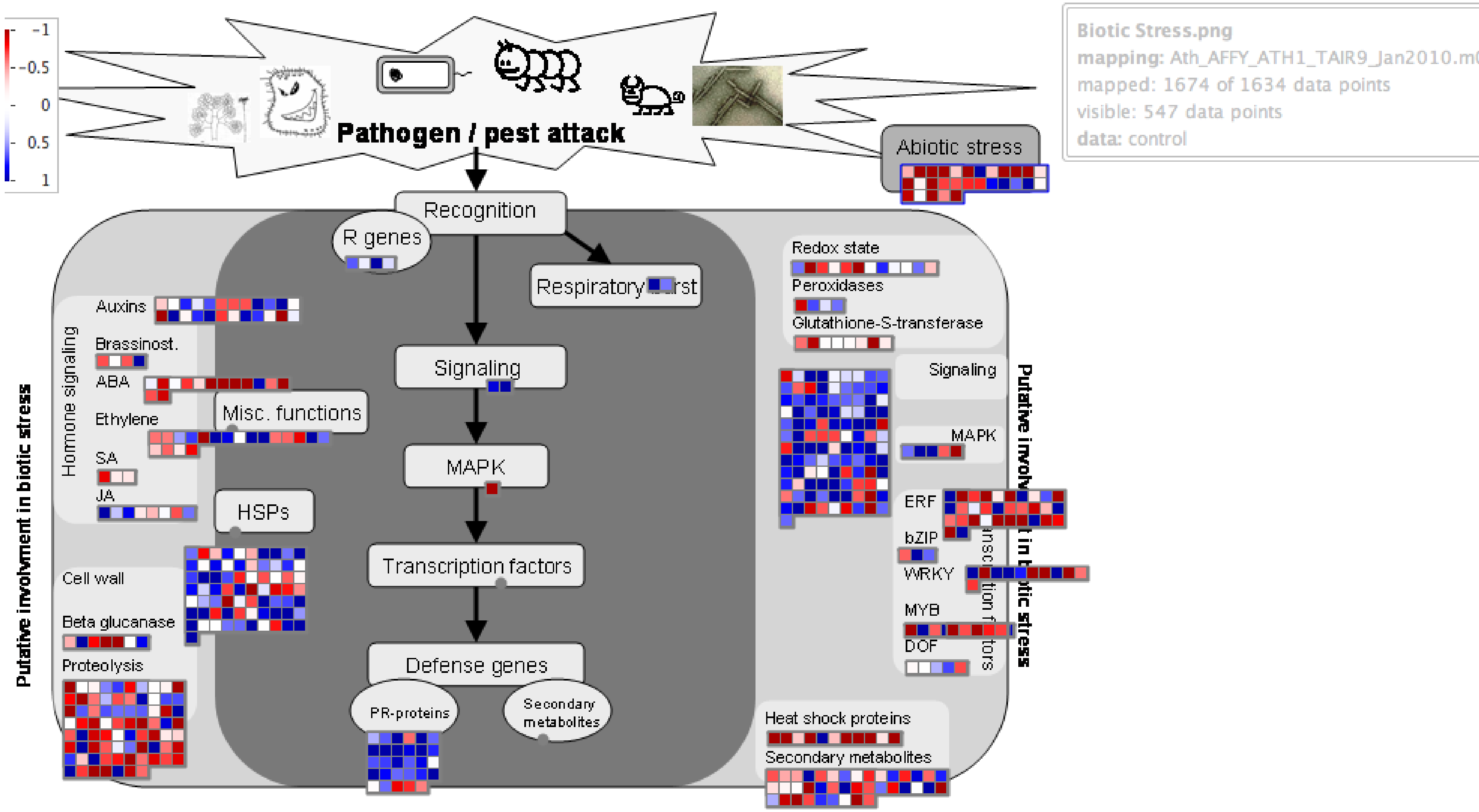

Supplementary Figure S7. Mapman analysis for *AtVIP2* overexpressor plants at 0 HAI for biotic stress.

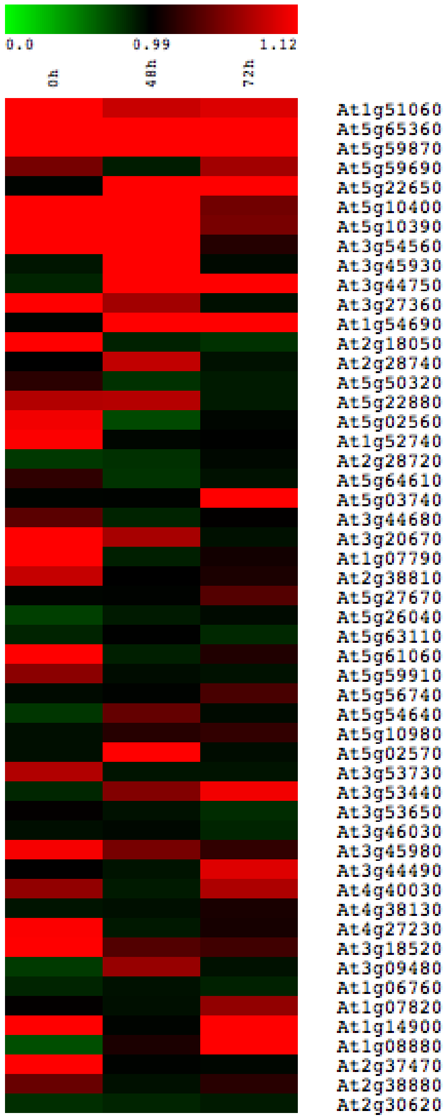

Supplementary Figure S8. Histones down-regulated in *Atvip2* mutant are up-regulated in *AtVIP2* overexpressors.

Supplementary Table S1: List of differentially expressed genes in *AtVIP2* overexpressor line compared to Col-0

Gene Identifier	Description	Ratio(VIP2OE /Col 0)	Ratio(Col48/Col0)	Ratio(Col72/ Col0)
At1g35140	Phosphate-responsive 1 family protein(PHI-1)	9.528976975	2.325848042	2.515897918
At3g30720	qua-quine starch(QQS)	9.326922158	2.615107497	1.494934013
At3g56400	WRKY DNA-binding protein 70(WRKY70)	5.151655092	0.822803607	2.884724429
At2g30500	Kinase interacting (KIP1-like) family protein(NET4B)	4.473451355	2.137241677	1.49055448
At3g45970	expansin-like A1(EXLA1)	3.870448239	1.996135189	1.795502724
At5g54380	receptor-like protein kinase THESEUS 1	3.400773238	1.485284659	2.558469297
At1g23030	ARM repeat superfamily protein(AT1G23030)	3.114264859	1.839732053	2.418163526
At4g02540	Cysteine/Histidine-rich C1 domain family protein(AT4G02540)	3.095395379	1.465489869	1.526906861
At5g24030	SLAC1 homologue 3(SLAH3)	3.089663081	1.929730376	3.624016731
At4g25810	xyloglucan endotransglycosylase 6(XTR6)	3.071495948	2.454203499	5.467729882
At4g01250	WRKY family transcription factor(WRKY22)	2.75570926	7.716544529	2.966252067
At1g15010	mediator of RNA polymerase II transcription subunit(AT1G15010)	2.71357324	4.726244816	2.335725137
At5g26920	Cam-binding protein 60-like G(CBP60G)	2.635920136	3.882528009	1.722785723
At5g57550	xyloglucan endotransglucosylase/hydrolase 25(XTH25)	2.584845928	22.20015136	16.15348124
At3g57930	rho GTPase-activating gacO-like protein(AT3G57930)	2.547173359	1.614994869	1.219579942
At1g50040	formin-like protein, putative (DUF1005)(AT1G50040)	2.467590209	4.480472862	2.401101368
At4g37610	BTB and TAZ domain protein 5(BT5)	2.363215343	3.111642644	1.784121192
At4g28085	transmembrane protein(AT4G28085)	2.352957682	4.394410541	2.683976242
At3g15770	hypothetical protein(AT3G15770)	2.194526686	8.334848749	2.889977299
At4g17460	Homeobox-leucine zipper protein 4 (HB-4) / HD-ZIP protein(HAT1)	2.177308718	1.970401237	1.21874166
At3g01430	NHL domain protein(AT3G01430)	2.148510598	4.833453254	3.185914386
At3g61190	BON association protein 1(BAP1)	2.102466401	2.175180541	1.611696091
At5g54610	ankyrin(ANK)	2.098059163	0.239694927	2.725641919
rpl16.mitochon	ribosomal protein L16	2.079899706	2.120296912	2.261118141
At5g24210	alpha/beta-Hydrolases superfamily protein(AT5G24210)	2.078823566	2.070890332	2.371450466
At3g44610	Protein kinase superfamily protein(AT3G44610)	2.035916172	1.794972924	1.279951596
At1g72430	SAUR-like auxin-responsive protein family(AT1G72430)	2.026118493	1.565986187	3.056615545
At3g15760	cytochrome P450 family protein(AT3G15760)	2.009467722	2.296923757	1.838831834
At5g54510	Auxin-responsive GH3 family protein(DFL1)	1.983410034	1.883444941	8.843018263

At4g19420	Pectinacetylesterase family protein(AT4G19420)	1.921426904	12.98987879	8.103346422
At5g01710	methyltransferase(AT5G01710)	1.880728257	1.510020407	1.324982127
At1g25390	Protein kinase superfamily protein(AT1G25390)	1.859243412	2.751943394	1.677222837
At4g22470	protease inhibitor/seed storage/lipid transfer protein (LTP) family protein(AT4G22470)	1.830174862	64.16823439	77.96290948
At4g23300	cysteine-rich RLK (RECEPTOR-like protein kinase) 22(CRK22)	1.57145125	5.205786894	4.798708532
At1g75040	pathogenesis-related protein 5(PR5)	0.537584996	0.14192146	0.318990091
At4g27570	UDP-Glycosyltransferase superfamily protein(AT4G27570)	0.525574998	0.071649954	0.072884554
At2g21650	Homeodomain-like superfamily protein(MEE3)	0.430796141	0.23967914	0.268153419
At1g10090	Early-responsive to dehydration stress protein (ERD4)(AT1G10090)	0.427389953	0.483474306	0.29335162
At3g23550	MATE efflux family protein(AT3G23550)	0.343286937	0.198736504	0.112883599
At1g20030	Pathogenesis-related thaumatin superfamily protein(AT1G20030)	0.332183322	0.311389845	0.339043188
At5g62360	Plant invertase/pectin methylesterase inhibitor superfamily protein(AT5G62360)	0.331807997	0.093802348	0.140368785
At5g50720	HVA22 homologue E(HVA22E)	0.331040486	0.612798418	0.37555309
At4g38410	Dehydrin family protein(AT4G38410)	0.325185697	0.140681012	0.245540458
At5g58770	Undecaprenyl pyrophosphate synthetase family protein(cPT4)	0.320720087	0.527414427	1.37869547
At1g64890	Major facilitator superfamily protein(AT1G64890)	0.314267694	0.235787415	0.247320629
At4g21990	APS reductase 3(APR3)	0.310213808	0.224342751	0.278093011
At5g24110	WRKY DNA-binding protein 30(WRKY30)	0.293060138	0.157882718	0.200107826
At5g57110	autoinhibited Ca2+ -ATPase(ACA8)	0.269312212	0.24541773	0.249476952
At1g68500	hypothetical protein(AT1G68500)	0.268992294	0.64666714	0.469494106
At1g53035	transmembrane protein(AT1G53035)	0.261673526	0.330245946	0.444903178
At1g32860	Glycosyl hydrolase superfamily protein(AT1G32860)	0.254304387	0.098004943	0.137293105
At3g13310	Chaperone DnaJ-domain superfamily protein(AT3G13310)	0.250617374	1.425269937	0.380140403
At1g26380	FAD-binding Berberine family protein(AT1G26380)	0.246488643	0.710658806	0.395973185
At2g39920	HAD superfamily, subfamily IIIB acid phosphatase(AT2G39920)	0.228593372	0.429448376	0.641255194
At3g26830	Cytochrome P450 superfamily protein(PAD3)	0.218558331	0.241249911	0.200279764
At2g19450	membrane bound O-acyl transferase (MBOAT) family protein(TAG1)	0.210537206	0.653541407	0.745189017
At3g05800	AtBS1(activation-tagged BRI1 suppressor 1)-interacting factor 1(AIF1)	0.203117366	0.13776453	0.098214968
At5g24470	two-component response regulator-like protein(PRR5)	0.142050168	0.555500475	0.42830841
At5g42900	cold regulated protein 27(COR27)	0.077437621	6.673125331	4.129613683
At5g20830	sucrose synthase 1(SUS1)	0.191927088	1.864699401	2.623607218
At2g43620	Chitinase family protein(AT2G43620)	0.255633256	4.199467716	1.741504375

At5g57630	CBL-interacting protein kinase 21(CIPK21)	0.272423615	2.176479673	1.475300412
At3g07650	CONSTANS-like 9(COL9)	0.277258003	3.761605952	2.458487058
At1g21110	O-methyltransferase family protein(IGMT3)	0.279131903	0.497503287	0.265243776
At4g33980	hypothetical protein(AT4G33980)	0.292338335	5.897793289	1.920562796
At5g04250	Cysteine proteinases superfamily protein(AT5G04250)	0.299945589	0.635866285	0.788757552
At5g54960	pyruvate decarboxylase-2(PDC2)	0.305054654	0.59682566	1.563292008
At5g23240	DNAJ heat shock N-terminal domain-containing protein(AT5G23240)	0.307990762	5.710547774	10.51207023
At3g49620	2-oxoglutarate (2OG) and Fe(II)-dependent oxygenase superfamily protein(DIN11)	0.325547352	2.297301167	1.132991211
At4g12470	azelaic acid induced 1(AZI1)	0.325771973	97.29920632	59.22651608
At2g47770	TSPO(outer membrane tryptophan-rich sensory protein)-like protein(TSPO)	0.334085865	5.471224586	8.359941281
At2g43590	Chitinase family protein(AT2G43590)	0.336589191	0.350603394	0.176187317
At1g65690	Late embryogenesis abundant (LEA) hydroxyproline-rich glycoprotein family(AT1G656	0.348133217	1.668093203	2.394019748
At2g31360	16:0delta9 desaturase 2(ADS2)	0.357660997	0.192930785	0.488727347
At5g25110	CBL-interacting protein kinase 25(CIPK25)	0.360040683	0.608924286	0.813472306
At1g21100	O-methyltransferase family protein(IGMT1)	0.361141426	0.677719398	0.267098543
At1g15520	pleiotropic drug resistance 12(ABCG40)	0.361880174	0.282947721	0.327663909
At1g76590	PLATZ transcription factor family protein(AT1G76590)	0.362965946	2.417581097	0.922868511
At3g12580	heat shock protein 70(HSP70)	0.364166968	0.548754981	3.334259945
At4g28140	Integrase-type DNA-binding superfamily protein(AT4G28140)	0.36443912	0.262218629	0.216223704
At2g40130	Double Clp-N motif-containing P-loop nucleoside triphosphate hydrolases superfamily	0.364498665	0.332692941	0.292536339
At2g40080	EARLY FLOWERING-like protein (DUF1313)(ELF4)	0.367586086	1.719161209	3.279293799
At1g21120	O-methyltransferase family protein(IGMT2)	0.37220022	0.574975512	0.357693592
At1g15670	Galactose oxidase/kelch repeat superfamily protein(AT1G15670)	0.37475504	6.546162245	2.9414434
At1g15100	RING-H2 finger A2A(RHA2A)	0.375312091	1.351623297	1.751027075
At2g15890	maternal effect embryo arrest 14(MEE14)	0.377149353	2.52128795	1.460980026
At1g48100	Pectin lyase-like superfamily protein(AT1G48100)	0.379714616	0.05525403	0.060639497
At4g34950	Major facilitator superfamily protein(AT4G34950)	0.380572523	0.466053511	1.547294199
At2g43570	chitinase(CHI)	0.38113162	3.289799552	5.863139389
At1g05100	mitogen-activated protein kinase kinase kinase 18(MAPKKK18)	0.38128014	0.793873151	2.879621608
At4g04330	Chaperonin-like RbcX protein(RbcX1)	0.381468044	0.064268446	0.375220931
At2g44130	Galactose oxidase/kelch repeat superfamily protein(AT2G44130)	0.38451899	1.573117853	0.653745821
At1g02820	Late embryogenesis abundant 3 (LEA3) family protein(LEA3)	0.38657808	0.209993321	0.485414435

At5g50450	HCP-like superfamily protein with MYND-type zinc finger(AT5G50450)	0.389731844	2.067783922	0.649897417
At5g47060	hypothetical protein (DUF581)(AT5G47060)	0.391603232	0.265543764	0.461131948
At5g52640	heat shock-like protein(HSP90.1)	0.392638567	1.309120493	4.746732306
At4g18170	WRKY DNA-binding protein 28(WRKY28)	0.394541796	2.130324245	1.874311246
At1g09350	galactinol synthase 3(GolS3)	0.394644832	0.050179763	0.038897976
At2g26560	phospholipase A 2A(PLA2A)	0.399594986	0.502600719	0.419900608
At5g61160	anthocyanin 5-aromatic acyltransferase 1(AACT1)	0.401008308	0.305847781	0.293814166
At5g05220	hypothetical protein(AT5G05220)	0.40646919	1.134147077	2.606822609
At4g11370	RING-H2 finger A1A(RHA1A)	0.406673447	0.652318407	0.471736762
At1g28330	dormancy-associated protein-like 1(DYL1)	0.407132627	3.789506484	1.755602163
At2g20880	Integrase-type DNA-binding superfamily protein(ERF53)	0.407872076	0.697219294	0.271777745
At1g51090	Heavy metal transport/detoxification superfamily protein(AT1G51090)	0.410914908	0.13968172	0.179268667
At1g62570	flavin-monooxygenase glucosinolate S-oxygenase 4(FMO GS-OX4)	0.410963073	0.337349216	0.384297825
At2g47890	B-box type zinc finger protein with CCT domain-containing protein(AT2G47890)	0.411535922	0.433407128	0.424819539
At3g21700	Ras-related small GTP-binding family protein(SGP2)	0.411693403	0.421865512	0.670983414
At3g55580	Regulator of chromosome condensation (RCC1) family protein(AT3G55580)	0.412382352	0.61939943	0.444448771
At1g77680	Ribonuclease II/R family protein(AT1G77680)	0.417251695	0.501734029	0.437847007
At2g35980	Late embryogenesis abundant (LEA) hydroxyproline-rich glycoprotein family(YLS9)	0.419931851	2.632835902	1.455579121
At1g68620	alpha/beta-Hydrolases superfamily protein(AT1G68620)	0.420975368	9.262234519	6.881914218
At2g27420	Cysteine proteinases superfamily protein(AT2G27420)	0.421783594	0.659858894	0.46258035
At4g34138	UDP-glucosyl transferase 73B1(UGT73B1)	0.422110865	4.506566548	2.114227992
At1g62180	5'adenylylphosphosulfate reductase 2(APR2)	0.425646723	0.322937676	0.797133514
At3g09540	Pectin lyase-like superfamily protein(AT3G09540)	0.425794099	0.076187767	0.103647196
At5g44210	erf domain protein 9(ERF9)	0.42605839	0.718215582	0.473680537
At2g15120	miscRNA(AT2G15120)	0.427942365	0.730952341	0.406769898
At1g78600	light-regulated zinc finger protein 1(LZF1)	0.428313101	0.110135981	0.331733072
At3g22600	Bifunctional inhibitor/lipid-transfer protein/seed storage 2S albumin superfamily protein	0.428477382	9.90384602	38.43299847
At3g16670	Pollen Ole e 1 allergen and extensin family protein(AT3G16670)	0.432816164	0.187794449	0.111116636
At1g62300	WRKY family transcription factor(WRKY6)	0.432817472	2.262995588	2.822434509
At4g28703	RmlC-like cupins superfamily protein(AT4G28703)	0.433780779	0.412815628	0.232652856
At5g48570	FKBP-type peptidyl-prolyl cis-trans isomerase family protein(AT5G48570)	0.433914351	0.252725259	0.692759462
At4g16146	cAMP-regulated phosphoprotein 19-related protein(AT4G16146)	0.434464506	0.564434293	0.85519962

At1g13740	ABI five binding protein 2(AFP2)	0.434816691	0.504683726	0.628055727
At5g55970	RING/U-box superfamily protein(AT5G55970)	0.439044774	1.96445819	0.518616649
At1g32870	NAC domain protein 13(NAC13)	0.439875561	0.636662898	1.015641231
At1g47710	Serine protease inhibitor (SERPIN) family protein(SERPIN1)	0.440595008	0.375610249	0.498745766
At3g57520	seed imbibition 2(SIP2)	0.442008303	2.856684071	1.507390226
At4g34135	UDP-glucosyltransferase 73B2(UGT73B2)	0.44218415	3.394993498	3.685750714
At1g46768	related to AP2 1(RAP2.1)	0.444647871	1.058623212	0.52469035
At1g10770	Plant invertase/pectin methylesterase inhibitor superfamily protein(AT1G10770)	0.445224944	0.49086385	0.410452423
At2g25930	hydroxyproline-rich glycoprotein family protein(ELF3)	0.447652742	2.463235793	1.43555425
At5g11150	vesicle-associated membrane protein 713(VAMP713)	0.450069068	1.930626522	1.91160284
At5g10760	Eukaryotic aspartyl protease family protein(AT5G10760)	0.450345273	0.478741126	1.17677413
At4g37400	cytochrome P450, family 81, subfamily F, polypeptide 3(CYP81F3)	0.450686687	0.235575865	0.238535035
At1g21790	TRAM, LAG1 and CLN8 (TLC) lipid-sensing domain containing protein(AT1G21790)	0.450864791	0.356508297	0.281644489
At1g64780	ammonium transporter 1;2(AMT1;2)	0.45304588	0.184649943	0.154779951
At1g48330	SsrA-binding protein(AT1G48330)	0.454906425	0.386687638	0.446946111
At2g30140	UDP-Glycosyltransferase superfamily protein(UGT87A2)	0.456664168	2.879550924	2.602733778
At5g37440	Chaperone DnaJ-domain superfamily protein(AT5G37440)	0.457378679	0.325737805	0.346509912
At1g16840	hypothetical protein(AT1G16840)	0.457704109	0.777019234	0.569066952
At1g07050	CCT motif family protein(AT1G07050)	0.459893832	0.622371957	1.050253679
At1g27630	cyclin T 1;3(CYCT1;3)	0.460159421	0.543007039	0.709092791
At1g01190	cytochrome P450, family 78, subfamily A, polypeptide 8(CYP78A8)	0.46119555	0.34098642	0.268019835
At3g50970	dehydrin family protein(LTI30)	0.461565861	0.508504228	0.383973897
At3g55760	hypothetical protein(AT3G55760)	0.462731396	0.102031352	0.141735605
At2g44940	Integrase-type DNA-binding superfamily protein(AT2G44940)	0.464289348	0.198295282	0.265954397
At1g77450	NAC domain containing protein 32(NAC032)	0.464519307	0.554908312	0.606149624
At4g01390	TRAF-like family protein(AT4G01390)	0.467795292	0.19704901	0.171950358
At3g04620	Alba DNA/RNA-binding protein(DAN1)	0.4707589	0.414809036	0.443054573
At1g10140	Uncharacterized conserved protein UCP031279(AT1G10140)	0.47161994	4.267829719	1.678727402
At1g78080	related to AP2 4(RAP2.4)	0.472176475	1.50523046	0.882641193
At4g16490	ARM repeat superfamily protein(AT4G16490)	0.472522097	0.48780894	0.450585498
At2g29460	glutathione S-transferase tau 4(GSTU4)	0.473540034	1.333410679	1.705339778
At2g46690	SAUR-like auxin-responsive protein family(AT2G46690)	0.47365194	2.95911125	2.680833115

At4g39780	Integrase-type DNA-binding superfamily protein(AT4G39780)	0.473958332	4.06348081	1.719451331
At4g38580	farnesylated protein 6(FP6)	0.474586764	0.799688414	0.400918819
At4g17550	Major facilitator superfamily protein(G3Pp4)	0.474927307	0.60695466	0.728576942
At1g26420	FAD-binding Berberine family protein(AT1G26420)	0.475457974	4.319386843	2.460232756
At2g28930	protein kinase 1B(PK1B)	0.475705279	0.568759873	0.598474448
At1g78170	E3 ubiquitin-protein ligase(AT1G78170)	0.477494427	0.632661162	0.418018648
At4g17090	chloroplast beta-amylase(CT-BMY)	0.47801346	0.253538663	0.565246847
At1g69870	nitrate transporter 1.7(NRT1.7)	0.480221311	0.272135824	0.443137832
At5g62920	response regulator 6(ARR6)	0.483952386	0.283873407	1.275578354
At4g01720	WRKY family transcription factor(WRKY47)	0.487039337	2.693506671	1.798553274
At1g24575	DEAD-box ATP-dependent RNA helicase-like protein(AT1G24575)	0.492709109	0.776820558	0.516957238
At5g47640	nuclear factor Y, subunit B2(NF-YB2)	0.493484698	0.496336004	0.349241133
At2g38080	Laccase/Diphenol oxidase family protein(IRX12)	0.493547936	0.238078628	0.211763551
At5g51570	SPFH/Band 7/PHB domain-containing membrane-associated protein family(AT5G515	0.494107803	3.533731639	3.280694531
At2g33830	Dormancy/auxin associated family protein(AT2G33830)	0.495876268	2.488751475	1.635019214
At1g51610	Cation efflux family protein(AT1G51610)	0.495976112	0.365834303	0.66195042
At1g16850	transmembrane protein(AT1G16850)	0.497004091	0.88150312	0.44002626
At1g76580	Squamosa promoter-binding protein-like (SBP domain) transcription factor family prote	0.497263588	0.546132913	0.307753555
At5g54120	unknown protein	0.49872554	1.751202182	0.790364711
At5g52300	CAP160 protein(LTI65)	0.498865421	1.730636108	2.032860363
At3g14560	hypothetical protein(AT3G14560)	0.498946987	2.795325429	1.817662429
At4g01026	PYR1-like 7(PYL7)	0.499988707	0.784484957	0.597359923
At2g23340	DREB and EAR motif protein 3(DEAR3)	0.501001992	0.593584257	0.496166017
At1g68050	flavin-binding, kelch repeat, f box 1(FKF1)	0.502070336	2.607786954	1.688800416
At3g05660	receptor like protein 33(RLP33)	0.502085046	0.162009054	0.148002084
At5g02020	E3 ubiquitin-protein ligase RLIM-like protein(SIS)	0.502143619	2.714755686	0.520783954
At4g20320	CTP synthase family protein(AT4G20320)	0.505489143	4.351347958	2.292891459
At1g27200	glycosyltransferase family protein (DUF23)(AT1G27200)	0.508468953	0.373094585	0.271541782
At1g79410	organic cation/carnitine transporter5(OCT5)	0.513047566	0.515188315	1.004854578
At2g19810	CCCH-type zinc finger family protein(OZF1)	0.513477412	1.601107107	0.618969627
At3g21690	MATE efflux family protein(AT3G21690)	0.515348621	1.61803197	2.79854431
At5g03240	polyubiquitin 3(UBQ3)	0.515995374	4.393439469	3.012698699

At2g28900	outer plastid envelope protein 16-1(OEP16-1)	0.516336272	0.207171014	0.195841366
At5g41410	POX (plant homeobox) family protein(BEL1)	0.517497308	1.647913603	1.878347713
At5g17760	P-loop containing nucleoside triphosphate hydrolases superfamily protein(AT5G17760	0.517861092	0.343412997	0.601300171
At4g15130	phosphorylcholine cytidylyltransferase2(CCT2)	0.517895652	0.438334688	0.368534588
At1g33230	TMPIT-like protein(AT1G33230)	0.518018775	0.542731751	0.387702525
At3g47160	RING/U-box superfamily protein(AT3G47160)	0.51824602	1.607237416	0.936706784
At1g12710	phloem protein 2-A12(PP2-A12)	0.518254492	0.693466318	0.621624938
At3g48360	BTB and TAZ domain protein 2(BT2)	0.519377444	3.655486428	2.536787442
At2g21620	Adenine nucleotide alpha hydrolases-like superfamily protein(RD2)	0.519773749	1.651558005	1.410172657
At1g69760	suppressor SRP40-like protein(AT1G69760)	0.520559809	1.479525554	2.994678963
At5g06570	alpha/beta-Hydrolases superfamily protein(AT5G06570)	0.521944775	8.79880985	4.256919777
At4g25480	dehydration response element B1A(DREB1A)	0.52249958	0.139542643	0.1761319
At3g56290	potassium transporter(AT3G56290)	0.523374246	0.284381404	0.706571322
At3g47800	Galactose mutarotase-like superfamily protein(AT3G47800)	0.523590822	2.374509443	1.282662439
At3g24520	heat shock transcription factor C1(HSFC1)	0.524043607	0.359810258	0.364965976
At3g61060	phloem protein 2-A13(PP2-A13)	0.524482192	3.981917656	1.565335126
At5g46050	peptide transporter 3(PTR3)	0.524918966	21.51598818	20.86580528
At1g78070	Transducin/WD40 repeat-like superfamily protein(AT1G78070)	0.525397349	0.444186073	0.291424114
At1g79160	filamentous hemagglutinin transporter(AT1G79160)	0.525872144	1.577585222	1.236092947
At5g60100	pseudo-response regulator 3(PRR3)	0.526083775	4.302647634	3.133815582
At1g11210	cotton fiber protein, putative (DUF761)(AT1G11210)	0.527583175	0.721209855	0.372712836
At5g63330	putative protein contains similarity to kinase	0.52765363	0.348554629	0.380525152
At5g25210	hypothetical protein(AT5G25210)	0.52933664	4.163519343	2.08172798
At2g02010	glutamate decarboxylase 4(GAD4)	0.529399134	0.376584073	0.448212602
At2g46310	cytokinin response factor 5(CRF5)	0.530183628	0.849380671	2.602958992
At4g38540	FAD/NAD(P)-binding oxidoreductase family protein(AT4G38540)	0.53028847	15.279162	5.662046454
At3g14440	nine-cis-epoxycarotenoid dioxygenase 3(NCED3)	0.531121413	0.401740302	0.191255349
At5g64100	Peroxidase superfamily protein(AT5G64100)	0.531563908	11.40481783	2.128829509
At4g24960	HVA22 homologue D(HVA22D)	0.5316028	0.145626765	0.14712218
At4g23450	RING/U-box superfamily protein(AIRP1)	0.532373717	2.18136079	1.232769741
At2g15320	Leucine-rich repeat (LRR) family protein(AT2G15320)	0.532556032	0.456444297	0.462431017
At1g49450	Transducin/WD40 repeat-like superfamily protein(AT1G49450)	0.533125595	2.191372936	1.650831393

At3g55450	PBS1-like 1(PBL1)	0.533705322	2.797246432	1.129691853
At4g29190	Zinc finger C-x8-C-x5-C-x3-H type family protein(OZF2)	0.534387251	1.392405186	0.57849901
At1g32900	UDP-Glycosyltransferase superfamily protein(GBSS1)	0.534652999	0.187306036	0.190656057
At5g40390	Raffinose synthase family protein(SIP1)	0.535759485	0.289620537	0.286426024
At3g04460	peroxin-12(PEX12)	0.536140828	1.028729371	0.635381933
At2g29720	FAD/NAD(P)-binding oxidoreductase family protein(CTF2B)	0.536911699	0.191989437	0.170076749
At1g74890	response regulator 15(ARR15)	0.537723531	0.154554577	0.169808549
At1g49230	RING/U-box superfamily protein(AT1G49230)	0.537830022	0.327539143	0.656714699
At3g06620	PAS domain-containing protein tyrosine kinase family protein(AT3G06620)	0.537959075	0.79547624	0.629322053
At1g08920	ERD (early response to dehydration) six-like 1(ESL1)	0.538103423	0.483639483	0.551094215
At3g17130	Plant invertase/pectin methylesterase inhibitor superfamily protein(AT3G17130)	0.5404075	0.142620459	0.156496386
At3g19260	LAG1 homologue 2(LOH2)	0.541889459	0.815693062	0.606497202
At3g16530	Legume lectin family protein(AT3G16530)	0.541963902	0.097848793	0.043042564
At1g02460	Pectin lyase-like superfamily protein(AT1G02460)	0.543650791	0.662185007	0.701301189
At2g18915	LOV KELCH protein 2(LKP2)	0.543781102	0.966289672	0.57690187
At5g09440	EXORDIUM like 4(EXL4)	0.545238713	5.242149895	3.361980511
At2g22020	hypothetical protein	0.545663492	0.598691958	0.395603969
At1g77000	RNI-like superfamily protein(SKP2B)	0.547385126	6.629391813	2.639159381
At5g43150	elongation factor(AT5G43150)	0.547775832	0.273500338	0.290865552
At1g73390	Endosomal targeting BRO1-like domain-containing protein(AT1G73390)	0.548080099	0.225654624	0.320386063
At5g06760	Late Embryogenesis Abundant 4-5(LEA4-5)	0.54827171	5.257337221	8.089834217
At2g22450	riboflavin biosynthesis protein(RIBA2)	0.549270342	1.920038198	1.558955871
At1g21540	AMP-dependent synthetase and ligase family protein(AT1G21540)	0.550377288	0.842999748	0.504832456
At3g12510	MADS-box family protein(AT3G12510)	0.550702124	0.882852617	2.001921741
At5g10170	myo-inositol-1-phosphate synthase 3(MIPS3)	0.551387197	0.223849376	0.219119823
At4g35630	phosphoserine aminotransferase(PSAT)	0.55339276	0.348613037	0.698256659
At1g01360	regulatory component of ABA receptor 1(RCAR1)	0.553488595	1.071291075	0.506146976
At3g50260	cooperatively regulated by ethylene and jasmonate 1(CEJ1)	0.554751803	0.663722903	0.3416339
At2g02710	PAS/LOV protein B(PLPB)	0.555136867	3.73201789	2.643872616
At3g55640	Mitochondrial substrate carrier family protein(AT3G55640)	0.555372808	0.533269988	0.440076125
At5g13180	NAC domain containing protein 83(NAC083)	0.5558927	1.258044903	1.638695903
At1g54010	GDSL-like Lipase/Acylhydrolase superfamily protein(AT1G54010)	0.556974482	0.15505647	0.139056808

At2g26020	plant defensin 1.2b(PDF1.2b)	0.557969717	0.336128607	0.219566745
At3g05220	Heavy metal transport/detoxification superfamily protein(AT3G05220)	0.558106421	0.512939047	0.507807894
At1g66830	Leucine-rich repeat protein kinase family protein(AT1G66830)	0.558152372	1.459772783	3.630124873
At2g43580	Chitinase family protein(AT2G43580)	0.558317756	0.50050204	0.418231469
At2g16350	hypothetical protein	0.559036186	0.419168055	0.628658563
At1g20070	hypothetical protein(AT1G20070)	0.559425445	0.398076648	0.649889239
At3g62770	Transducin/WD40 repeat-like superfamily protein(AtATG18a)	0.560132611	1.546147445	0.92614768
At2g31940	oxidoreductase/transition metal ion-binding protein(AT2G31940)	0.561503536	0.774737075	0.480832865
At5g11890	harpin-induced protein(EMB3135)	0.562271769	0.328042226	0.177049165
At2g38210	putative PDX1-like protein 4(PDX1L4)	0.563135358	1.886282388	1.170125324
At5g14570	high affinity nitrate transporter 2.7(NRT2.7)	0.564297872	0.591785157	0.400255095
At3g61630	cytokinin response factor 6(CRF6)	0.565690802	1.397977232	0.601247762
At4g13250	NAD(P)-binding Rossmann-fold superfamily protein(NYC1)	0.565779718	3.365425704	2.663185616
At5g61520	Major facilitator superfamily protein(AT5G61520)	0.566945858	3.420585002	2.203250929
At1g32960	Subtilase family protein(SBT3.3)	0.567233159	0.467325272	0.585491769
At4g32290	Core-2/I-branching beta-1,6-N-acetylglucosaminyltransferase family protein(AT4G322	0.567614408	0.538665637	0.29798345
At5g61820	stress up-regulated Nod 19 protein(AT5G61820)	0.56792414	1.711081739	2.073082608
At4g23920	UDP-D-glucose/UDP-D-galactose 4-epimerase 2(UGE2)	0.56806179	0.401055169	0.401321969
At1g49560	Homeodomain-like superfamily protein(AT1G49560)	0.568579017	0.548407395	0.817624999
At1g21130	O-methyltransferase family protein(IGMT4)	0.569014879	0.519832105	0.332245547
At1g52200	PLAC8 family protein(AT1G52200)	0.569055131	4.151639535	2.882718942
At2g46170	Reticulon family protein(AT2G46170)	0.569494971	0.248436272	0.539551563
At3g57240	beta-1,3-glucanase 3(BG3)	0.56954081	0.225902162	0.201538717
At3g61890	homeobox 12(HB-12)	0.569900611	4.288272869	1.646677875
At1g10410	CW14 protein (DUF1336)(AT1G10410)	0.569904727	1.574461686	1.263799119
At3g60980	Tetratricopeptide repeat (TPR)-like superfamily protein(AT3G60980)	0.570034326	0.597465442	0.665363672
At2g16700	actin depolymerizing factor 5(ADF5)	0.571367461	1.211303876	1.785464134
At5g09400	K+ uptake permease 7(KUP7)	0.571558168	1.576730587	1.135827519
At1g24600	hypothetical protein(AT1G24600)	0.572490593	4.8061538	6.923436579
At2g38820	DNA-directed RNA polymerase subunit beta-beta protein, putative (DUF506)(AT2G38	0.572601044	3.521833373	1.116361082
At1g19370	membrane protein(AT1G19370)	0.574353855	0.598709669	0.64915934
At4g14230	CBS domain protein with a domain protein (DUF21)(AT4G14230)	0.574567274	0.597594731	0.787294277

At2g24240	BTB/POZ domain with WD40/YVTN repeat-like protein(AT2G24240)	0.575361805	3.200071235	1.583743922
At5g52450	MATE efflux family protein(AT5G52450)	0.575389207	4.632204258	3.84364614
At3g15500	NAC domain containing protein 3(NAC3)	0.575409998	1.783389318	1.23026534
At2g36470	DUF868 family protein, putative (DUF868)(AT2G36470)	0.57680729	0.819486612	0.39052651
At5g17490	RGA-like protein 3(RGL3)	0.578505884	0.346410272	0.144292734
At3g09390	metallothionein 2A(MT2A)	0.57877101	1.804032148	1.988629662
At3g24070	Zinc knuckle (CCHC-type) family protein(AT3G24070)	0.579243408	3.395165134	1.578529896
At3g17790	purple acid phosphatase 17(PAP17)	0.579858047	6.058138164	4.171308083
At1g02360	Chitinase family protein(AT1G02360)	0.580775927	0.676822156	0.45341156
At1g22690	Gibberellin-regulated family protein(AT1G22690)	0.581144144	0.055071327	0.042681091
At4g19810	Glycosyl hydrolase family protein with chitinase insertion domain-containing protein(Cł	0.581278999	1.971858488	1.705292433
At3g09410	Pectinacetylesterase family protein(AT3G09410)	0.582015453	0.43994389	0.326546696
At4g21510	F-box family protein(FBS2)	0.582418576	1.805162462	1.113798827
At4g13710	Pectin lyase-like superfamily protein(AT4G13710)	0.582577912	0.338399994	0.400785785
At5g50200	nitrate transmembrane transporter(WR3)	0.582638388	16.55910161	16.22884694
At5g24570	hypothetical protein(AT5G24570)	0.583311514	0.605219529	0.792596079
At1g75810	transmembrane protein(AT1G75810)	0.583490726	2.024029636	1.642958653
At4g26670	Mitochondrial import inner membrane translocase subunit Tim17/Tim22/Tim23 family p	0.584578201	0.528815759	0.88686774
At3g16500	phytochrome-associated protein 1(PAP1)	0.585113781	0.598175299	0.540673329
At1g14720	xyloglucan endotransglucosylase/hydrolase 28(XTH28)	0.585743998	0.63216593	1.119003681
At4g34560	transmembrane protein(AT4G34560)	0.586248002	0.248406728	0.341656546
At5g05790	Duplicated homeodomain-like superfamily protein(AT5G05790)	0.587111074	0.704372445	0.393433185
At3g07080	EamA-like transporter family(AT3G07080)	0.587452201	0.644836973	0.885292561
At5g39760	homeobox protein 23(HB23)	0.587669186	0.459016429	0.834300385
At2g47190	myb domain protein 2(MYB2)	0.588371882	10.30463812	7.261741216
At1g35230	arabinogalactan protein 5(AGP5)	0.589338238	0.634812599	1.021924824
At5g04220	Calcium-dependent lipid-binding (CaLB domain) family protein(SYTC)	0.589520994	2.854179542	2.466187034
At5g38120	AMP-dependent synthetase and ligase family protein(4CL8)	0.589907229	0.231551815	0.209798266
At5g62450	unknown protein	0.590624809	0.615199858	1.076539346
At2g38465	hypothetical protein(AT2G38465)	0.591423357	3.977512767	3.477026813
At5g12110	elongation factor 1-beta 1(AT5G12110)	0.59181018	0.157001558	0.143933692
At4g30650	Low temperature and salt responsive protein family(AT4G30650)	0.591964227	0.184137024	0.195818021

At5g47450	tonoplast intrinsic protein 2;3(TIP2;3)	0.591992907	4.495558274	1.598229938
At3g16920	chitinase-like protein(CTL2)	0.592045799	0.262248658	0.23537486
At2g22800	Homeobox-leucine zipper protein family(HAT9)	0.592108041	2.422192326	0.91715554
At5g01210	HXXXD-type acyl-transferase family protein(AT5G01210)	0.59235328	0.448472856	0.176780061
At1g76800	Vacuolar iron transporter (VIT) family protein(AT1G76800)	0.592944886	0.590600274	0.475608549
At4g01870	tolB protein-like protein(AT4G01870)	0.593418928	1.853699653	1.116585073
At4g20780	calmodulin like 42(CML42)	0.594623881	0.304116821	0.309671178
At2g42530	cold regulated 15b(COR15B)	0.594671888	0.119680575	0.22825679
At4g28150	hypothetical protein (DUF789)(AT4G28150)	0.595097392	0.560143583	0.414640257
At1g55500	evolutionarily conserved C-terminal region 4(ECT4)	0.595679429	0.577641861	1.189423092
At2g39400	alpha/beta-Hydrolases superfamily protein(AT2G39400)	0.596326125	1.725112553	0.71482966
At3g18830	polyol/monosaccharide transporter 5(PMT5)	0.599136503	0.654548307	0.912960973
At1g68520	B-box type zinc finger protein with CCT domain-containing protein(BBX14)	0.599351093	2.492432389	2.876745973
At5g01600	ferretin 1(FER1)	0.599897765	1.470099347	2.030561274
At1g64710	GroES-like zinc-binding alcohol dehydrogenase family protein(AT1G64710)	0.600796671	0.1824216	0.143375162
At2g26980	CBL-interacting protein kinase 3(CIPK3)	0.600812438	1.080812267	2.206061599
At2g18350	homeobox protein 24(HB24)	0.600856183	0.586658459	0.564060614
At2g36320	A20/AN1-like zinc finger family protein(AT2G36320)	0.601324829	2.668573476	1.500351154
At5g62440	EMB514 (DUF3223)(AT5G62440)	0.60144993	0.870168074	1.573308744
At1g78310	VQ motif-containing protein(AT1G78310)	0.602322352	2.435330966	1.303041433
At4g26850	GDP-L-galactose phosphorylase 1(VTC2)	0.602875727	0.896303803	2.181569516
At3g05640	Protein phosphatase 2C family protein(AT3G05640)	0.602963717	1.475726646	0.49862495
At5g60790	ABC transporter family protein(ABCF1)	0.60304757	0.396034209	0.81047848
At1g79660	ephrin-A3 protein(AT1G79660)	0.603061787	0.607277104	0.420558609
At5g19875	transmembrane protein(AT5G19875)	0.603159458	0.537803969	0.593659066
At5g26220	ChaC-like family protein(AT5G26220)	0.603477252	0.546416797	0.525286776
At1g36370	serine hydroxymethyltransferase 7(SHM7)	0.603666159	0.543653113	0.590882355
At1g01500	Erythronate-4-phosphate dehydrogenase family protein(AT1G01500)	0.603791433	0.617030503	0.830562628
At5g28150	hypothetical protein (DUF868)(AT5G28150)	0.604157545	0.277402312	0.463985165
At5g06690	WCRKC thioredoxin 1(WCRKC1)	0.604434452	1.801013615	0.878643929
At4g27070	tryptophan synthase beta-subunit 2(TSB2)	0.60458352	0.702907593	0.44799281
At3g25890	Integrase-type DNA-binding superfamily protein(CRF11)	0.604771374	0.350704621	1.277227933

At1g19050	response regulator 7(ARR7)	0.604979237	0.277719176	1.072580667
At3g19290	ABRE binding factor 4(ABF4)	0.605431303	2.30941415	1.41443699
At3g57540	Remorin family protein(AT3G57540)	0.605465512	0.792085931	0.66035595
At1g71960	ATP-binding casette family G25(ABCG25)	0.606088218	0.598637224	0.685069282
At4g24240	WRKY DNA-binding protein 7(WRKY7)	0.607637907	2.206072832	1.804822543
At1g78450	SOUL heme-binding family protein(AT1G78450)	0.608222617	0.882863737	0.598823062
At2g37980	O-fucosyltransferase family protein(AT2G37980)	0.608491747	0.901073447	0.632366906
At3g59940	Galactose oxidase/kelch repeat superfamily protein(AT3G59940)	0.609392629	2.500632061	1.627861221
At1g56600	galactinol synthase 2(GolS2)	0.60959401	0.225324185	0.137359888
At4g36010	Pathogenesis-related thaumatin superfamily protein(AT4G36010)	0.609646988	0.068509083	0.129272862
At3g44990	xyloglucan endo-transglycosylase-related 8(XTH31)	0.609776063	0.350976304	0.270269001
At2g22080	transmembrane protein(AT2G22080)	0.611457225	1.340770215	0.498111332
At1g13950	eukaryotic elongation factor 5A-1(ELF5A-1)	0.611965111	1.196394365	1.533706763
At4g27520	early nodulin-like protein 2(ENODL2)	0.6120662	0.13283978	0.281682884
At3g15720	Pectin lyase-like superfamily protein(AT3G15720)	0.612696451	0.484675454	3.055726376
At3g22060	Receptor-like protein kinase-related family protein(AT3G22060)	0.612886626	0.848781569	0.481655894
At4g03430	pre-mRNA splicing factor-like protein(EMB2770)	0.61393241	0.514381284	0.579683375
At5g37260	Homeodomain-like superfamily protein(RVE2)	0.614087635	6.405369848	7.850561831
At2g24500	Zinc finger protein 622(FZF)	0.614208518	0.60547641	0.94365819
At5g58700	phosphatidylinositol-speciwc phospholipase C4(PLC4)	0.614292362	0.307355539	0.401616731
At4g04610	APS reductase 1(APR1)	0.614373883	0.143652993	0.173076917
At1g29395	COLD REGULATED 314 INNER MEMBRANE 1(COR413IM1)	0.614688049	1.525602993	0.388530029
At1g80110	phloem protein 2-B11(PP2-B11)	0.616332261	1.535745042	0.97229943
At4g00410	putative protein	0.616410558	0.380181592	0.537120129
At1g43860	sequence-specific DNA binding transcription factor(AT1G43860)	0.616917685	0.64224375	0.841595282
At4g21120	amino acid transporter 1(AAT1)	0.617568134	19.33233097	12.83656273
At4g12490	Bifunctional inhibitor/lipid-transfer protein/seed storage 2S albumin superfamily protein	0.617574998	137.9048402	132.0637563
At1g77120	alcohol dehydrogenase 1(ADH1)	0.618399774	4.363170268	7.086877337
At5g14760	L-aspartate oxidase(AO)	0.618465695	0.432796596	0.541578732
At2g13960	Homeodomain-like superfamily protein(AT2G13960)	0.618775047	1.819687644	0.708982753
At5g67190	DREB and EAR motif protein 2(DEAR2)	0.618776185	0.792751748	0.424552021
At2g45560	cytochrome P450, family 76, subfamily C, polypeptide 1(CYP76C1)	0.619173019	0.249960929	0.808840082

At2g28550	related to AP2.7(RAP2.7)	0.619380267	0.231912556	0.346958248
At1g69270	receptor-like protein kinase 1(RPK1)	0.619893621	1.580471763	1.034522667
At2g43020	polyamine oxidase 2(PAO2)	0.619899825	0.277736829	0.470455375
At1g01390	UDP-Glycosyltransferase superfamily protein(AT1G01390)	0.619912104	0.448395092	0.510978898
At5g65660	hydroxyproline-rich glycoprotein family protein(AT5G65660)	0.620089878	0.849465518	2.387237023
At1g62050	Ankyrin repeat family protein(AT1G62050)	0.620306834	0.365137146	0.674630549
At3g04630	WVD2-like 1(WDL1)	0.621354006	0.118305	0.064514761
At1g26770	expansin A10(EXPA10)	0.621707634	0.506076015	0.695864677
At5g55700	beta-amylase 4(BAM4)	0.622190667	2.734933266	0.749636151
At3g50410	OBF binding protein 1(OBP1)	0.623760381	0.513005428	0.792585411
At4g23630	VIRB2-interacting protein 1(BTI1)	0.625045896	0.550464581	0.738718559
At3g56090	ferritin 3(FER3)	0.625307541	0.1375604	0.559757043
At1g70000	myb-like transcription factor family protein(AT1G70000)	0.625567874	0.455428141	0.728453937
At1g49470	transmembrane epididymal protein (DUF716)(AT1G49470)	0.625761571	1.716839318	2.494355593
At1g79680	WALL ASSOCIATED KINASE (WAK)-LIKE 10(WAKL10)	0.626446235	0.503719678	0.36961182
At2g03760	sulfotransferase 12(SOT12)	0.62698617	1.461627156	1.684182773
At1g65500	transmembrane protein(AT1G65500)	0.627306827	8.606328428	8.613429827
At2g40180	phosphatase 2C5(PP2C5)	0.627322447	0.483828973	0.468681732
At5g59220	PP2C protein (Clade A protein phosphatases type 2C)(HAI1)	0.627582839	1.327236753	1.647253472
At3g15450	aluminum induced protein with YGL and LRDR motifs(AT3G15450)	0.627766664	2.04247058	1.274519553
At5g48180	nitrile specifier protein 5(NSP5)	0.627901514	13.1827427	12.52905572
At2g38560	transcript elongation factor IIS(TFIIS)	0.628479104	1.591342782	1.039180811
At5g51680	hydroxyproline-rich glycoprotein family protein(AT5G51680)	0.628718953	2.403065505	1.157815223
At4g12410	SAUR-like auxin-responsive protein family(AT4G12410)	0.628920121	0.333008995	0.405088219
At1g17050	solanesyl diphosphate synthase 2(SPS2)	0.629182819	0.201124072	0.488584305
At1g64105	NAC domain containing protein 27(NAC027)	0.629257796	0.586335956	0.810113048
At1g48210	Protein kinase superfamily protein(AT1G48210)	0.630340888	1.868835748	1.662684536
At1g64370	filaggrin-like protein(AT1G64370)	0.631035464	0.280478382	0.380484712
At5g53710	hypothetical protein(AT5G53710)	0.631380297	1.889953026	2.220537165
At4g27820	beta glucosidase 9(BGLU9)	0.631437872	0.360389178	1.00604155
At3g61900	SAUR-like auxin-responsive protein family(AT3G61900)	0.631542023	11.19001952	5.632253964
At5g60020	laccase 17(LAC17)	0.633057212	0.300461388	0.255802151

At2g40240	Tetratricopeptide repeat (TPR)-like superfamily protein(AT2G40240)	0.63351896	0.559725315	0.855564945
At1g70900	hypothetical protein(AT1G70900)	0.633572435	0.587363277	0.533654297
At3g24840	Sec14p-like phosphatidylinositol transfer family protein(AT3G24840)	0.633840835	0.57522952	0.56865458
At3g62690	AtL5(ATL5)	0.63420703	0.543421857	0.547494272
At3g24503	aldehyde dehydrogenase 2C4(ALDH2C4)	0.634568152	0.525626121	0.393298425
At4g11360	RING-H2 finger A1B(RHA1B)	0.634568853	3.449764263	1.762588347
At2g47180	galactinol synthase 1(GolS1)	0.635458263	1.071851239	0.597247362
At3g23000	CBL-interacting protein kinase 7(CIPK7)	0.635702924	0.168712092	0.439685223
At5g23510	hypothetical protein(AT5G23510)	0.635915606	0.675819376	0.580925388
At1g07280	Tetratricopeptide repeat (TPR)-like superfamily protein(AT1G07280)	0.63739793	0.625915471	0.800267277
At5g37500	gated outwardly-rectifying K+ channel(GORK)	0.637641983	3.012201036	3.084389468
At3g63060	EID1-like 3(EDL3)	0.63783946	0.762990773	1.859614659
At2g18170	MAP kinase 7(MPK7)	0.638353688	1.9286305	1.910719319
At2g43160	ENTH/VHS family protein(AT2G43160)	0.638366368	1.8367254	0.940590732
At1g67360	Rubber elongation factor protein (REF)(AT1G67360)	0.639500671	0.406932931	0.818508162
At4g33540	metallo-beta-lactamase family protein(AT4G33540)	0.640176925	4.170333665	4.049962352
At2g35700	ERF family protein 38(ERF38)	0.640494872	0.397736434	0.462441626
At5g57220	cytochrome P450, family 81, subfamily F, polypeptide 2(CYP81F2)	0.640776514	0.396739517	0.268751251
At3g62660	galacturonosyltransferase-like 7(GATL7)	0.641249288	0.662452092	0.635707058
At3g02230	reversibly glycosylated polypeptide 1(RGP1)	0.641694219	0.575380773	0.548482795
At1g01030	AP2/B3-like transcriptional factor family protein(NGA3)	0.64218452	0.47194722	0.460044077
At1g22985	Integrase-type DNA-binding superfamily protein(CRF7)	0.642323985	2.233296269	1.160437299
At1g05270	TraB family protein(AT1G05270)	0.642437008	2.189288561	1.072453953
At4g25500	arginine/serine-rich splicing factor 35(RS40)	0.642691094	2.636568408	1.132662112
At2g27820	prephenate dehydratase 1(PD1)	0.643086037	0.327063729	0.653738411
At1g20696	high mobility group B3(HMGB3)	0.643791706	1.744446957	1.656593325
At3g51110	Tetratricopeptide repeat (TPR)-like superfamily protein(AT3G51110)	0.644746328	3.931100194	1.811038642
At4g27830	beta glucosidase 10(BGLU10)	0.645086132	1.398575339	1.849163907
At5g03720	heat shock transcription factor A3(HSFA3)	0.645223359	0.58843143	0.476515753
At5g25280	serine-rich protein-like protein(AT5G25280)	0.645790739	3.372653542	2.778163986
At2g31230	ethylene-responsive element binding factor 15(ERF15)	0.645895639	0.397066806	0.664726932
At3g19930	sugar transporter 4(STP4)	0.646713285	1.770658095	1.037693324

At4g12000	SNARE associated Golgi protein family(AT4G12000)	0.646736204	0.354721988	0.316169785
At2g47140	NAD(P)-binding Rossmann-fold superfamily protein(SDR5)	0.646786983	0.660149725	0.559569336
At4g18520	Pentatricopeptide repeat (PPR) superfamily protein(AT4G18520)	0.646840266	0.370134052	0.691103381
At1g70300	K+ uptake permease 6(KUP6)	0.647361086	2.208815432	2.861956035
At5g01410	Aldolase-type TIM barrel family protein(RSR4)	0.647381439	0.358511944	0.557265388
At4g34230	cinnamyl alcohol dehydrogenase 5(CAD5)	0.647460947	1.906969379	4.381097277
At3g47430	peroxin 11B(PEX11B)	0.64774466	1.044954096	1.97862099
At2g40900	nodulin MtN21 /EamA-like transporter family protein(UMAMIT11)	0.647822929	0.37358405	0.366120448
At3g18560	hypothetical protein(AT3G18560)	0.648596413	4.208709543	1.507621403
At5g43260	chaperone protein dnaJ-like protein(AT5G43260)	0.648613129	1.474478809	1.937692372
At5g55420	miscRNA(AT5G55420)	0.648716842	0.509523919	0.498769296
At5g57280	S-adenosyl-L-methionine-dependent methyltransferases superfamily protein(RID2)	0.64923804	0.452092721	1.050108571
At1g21670	DPP6 amino-terminal domain protein(AT1G21670)	0.649243414	2.029509137	1.797872726
At2g23840	HNH endonuclease(AT2G23840)	0.650260824	0.297334483	0.811935995
At3g60520	zinc ion-binding protein(AT3G60520)	0.651498852	0.257458643	0.254009363
At1g55920	serine acetyltransferase 2;1(SERAT2;1)	0.651841779	0.794582636	1.510016388
At2g20680	Glycosyl hydrolase superfamily protein(MAN2)	0.652151231	0.879486582	0.402238818
At4g36670	Major facilitator superfamily protein(PMT6)	0.652800376	2.740103729	2.732862878
At1g55210	Disease resistance-responsive (dirigent-like protein) family protein(AT1G55210)	0.6536104	0.134636552	0.57260733
At5g23850	O-glucosyltransferase rumi-like protein (DUF821)(AT5G23850)	0.653844823	0.421905445	0.460665227
At1g72770	HYPERSENSITIVE TO ABA1(HAB1)	0.654227908	1.812940273	1.463012821
At5g54470	B-box type zinc finger family protein(BBX29)	0.655098952	1.152916108	0.38720247
At5g59340	WUSCHEL related homeobox 2(WOX2)	0.6553797	0.535325829	0.739571575
At1g13990	plant/protein(AT1G13990)	0.655732253	4.158916266	2.813315737
At4g24040	trehalase 1(TRE1)	0.655816898	5.828237197	3.158962167
At5g60540	pyridoxine biosynthesis 2(PDX2)	0.656046737	1.390298293	1.535139155
At2g02930	glutathione S-transferase F3(GSTF3)	0.65608624	2.144255936	0.897340801
At3g03640	beta glucosidase 25(BGLU25)	0.656433536	0.518113179	0.493828863
At4g36900	related to AP2 10(RAP2.10)	0.656540706	1.195250125	0.637607495
At2g16900	phospholipase-like protein (PEARLI 4) family protein(AT2G16900)	0.657565346	0.580615386	0.591323768
At1g78100	F-box family protein(AUF1)	0.657943649	0.648812274	0.743526242
At5g65860	ankyrin repeat family protein(AT5G65860)	0.658625039	0.349798938	0.571710885

At5g23050	acyl-activating enzyme 17(AAE17)	0.658913856	17.92490242	5.938445009
At2g40475	hypothetical protein(ASG8)	0.659531721	0.227597798	0.276988211
At4g32340	Tetratricopeptide repeat (TPR)-like superfamily protein(AT4G32340)	0.660014753	2.713438091	2.420158137
At5g03080	Phosphatidic acid phosphatase (PAP2) family protein(LPPgamma)	0.660297669	0.482779552	0.62704393
At5g43450	2-oxoglutarate (2OG) and Fe(II)-dependent oxygenase superfamily protein(AT5G4345	0.660350381	1.422100624	1.784318505
At4g27030	fatty acid desaturase A(FADA)	0.660432702	0.158366971	0.530011928
At1g62710	beta vacuolar processing enzyme(BETA-VPE)	0.660746539	0.170677071	0.35602901
At4g14990	Topoisomerase II-associated protein PAT1(AT4G14990)	0.660798557	2.015660142	1.087280718
At5g17600	RING/U-box superfamily protein(AT5G17600)	0.660833822	0.335017349	0.53208117
At4g33700	CBS domain protein (DUF21)(AT4G33700)	0.661118798	1.487179205	2.347665399
At2g03340	WRKY DNA-binding protein 3(WRKY3)	0.661224841	1.920195268	2.246979777
At4g34740	GLN phosphoribosyl pyrophosphate amidotransferase 2(ASE2)	0.66298326	0.306796113	0.79189533
At5g38510	Rhomboid-related intramembrane serine protease family protein(AT5G38510)	0.663901681	0.417291393	1.21308535
At3g49430	SER/ARG-rich protein 34A(SR34a)	0.664018348	0.455134032	0.471306524
At2g39710	Eukaryotic aspartyl protease family protein(AT2G39710)	0.66489225	3.45868122	6.663391295
At5g51180	alpha/beta-Hydrolases superfamily protein(AT5G51180)	0.66541003	0.920213477	0.597778943
At1g78995	hypothetical protein(AT1G78995)	0.665487609	0.287731633	0.685862611
At2g29670	Tetratricopeptide repeat (TPR)-like superfamily protein(AT2G29670)	0.665520081	1.636352921	0.557532994
At5g24320	Transducin/WD40 repeat-like superfamily protein(AT5G24320)	0.665627253	1.559785553	1.152979111
At5g57655	xylose isomerase family protein(AT5G57655)	0.665739351	8.78543845	4.382576296
At1g67920	hypothetical protein(AT1G67920)	0.666179551	1.537884773	0.94593387
At2g15960	stress-induced protein(AT2G15960)	0.666250761	2.131840964	1.769748397
At2g47060	Protein kinase superfamily protein(PTI1-4)	1.500314583	0.461713003	0.278885861
At5g04320	Shugoshin C terminus(AT5G04320)	1.500878938	0.591210763	0.695765932
At2g47270	transcription factor UPBEAT protein(UPB1)	1.50168615	13.52255589	5.930678177
At4g28400	Protein phosphatase 2C family protein(AT4G28400)	1.503186135	0.745456912	0.583545585
At4g27652	hypothetical protein(AT4G27652)	1.503566114	0.20244135	0.322004125
At1g77800	PHD finger family protein(AT1G77800)	1.50362387	2.088146796	1.95073026
At3g17680	Kinase interacting (KIP1-like) family protein(AT3G17680)	1.504280078	0.731436697	0.573251063
At2g34620	Mitochondrial transcription termination factor family protein(AT2G34620)	1.504429735	0.179524539	0.315458442
At5g01030	enolase, putative (DUF3527)(AT5G01030)	1.504553297	1.56202678	1.192785797
At2g30790	photosystem II subunit P-2(PSBP-2)	1.505301325	1.801692447	1.10017462

At1g74380	xyloglucan xylosyltransferase 5(XXT5)	1.50548899	0.446681886	0.504782687
At3g28220	TRAF-like family protein(AT3G28220)	1.507365706	0.184221481	0.168404506
At1g64060	respiratory burst oxidase protein F(RBOH F)	1.508044898	1.495896506	1.715438604
At2g34470	urease accessory protein G(UREG)	1.509287204	1.658865145	1.391774605
At1g76240	DUF241 domain protein (DUF241)(AT1G76240)	1.510912193	0.594118685	0.791801815
At5g44020	HAD superfamily, subfamily IIIB acid phosphatase(AT5G44020)	1.511078777	3.497038704	2.479052085
At2g24630	Glycosyl transferase family 2 protein(ATCSLC08)	1.512040251	0.647538244	0.74030795
At5g61440	atypical CYS HIS rich thioredoxin 5(ACHT5)	1.512988586	1.960473894	1.710659039
At4g12730	FASCICLIN-like arabinogalactan 2(FLA2)	1.514668366	0.113835726	0.091198067
At1g66940	kinase-like protein(AT1G66940)	1.5161726	0.224972036	0.126746808
At1g54040	epithiospecifier protein(ESP)	1.51668683	0.173173005	0.116172389
At5g58930	hypothetical protein (DUF740)(AT5G58930)	1.516766396	0.478784337	0.743301929
At4g12970	stomagen(STOMAGEN)	1.518076899	0.182324617	0.216285231
At1g53840	pectin methylesterase 1(PME1)	1.519243171	0.495406737	0.613114169
At1g28380	MAC/Perforin domain-containing protein(NSL1)	1.520237249	0.766755896	0.641484505
At4g12720	MutT/nudix family protein(NUDT7)	1.520739114	0.543877478	0.525491497
At4g28080	Tetratricopeptide repeat (TPR)-like superfamily protein(AT4G28080)	1.520826084	0.2861661	0.545315356
At4g09550	AtGCP3 interacting protein 1(GIP1)	1.522144431	0.458824581	0.566838159
At5g39580	Peroxidase superfamily protein(AT5G39580)	1.522823387	49.31524449	20.06436795
At3g54560	histone H2A 11(HTA11)	1.52299307	0.126831628	0.098946442
At5g51100	Fe superoxide dismutase 2(FSD2)	1.523215191	0.533651492	0.484396053
At2g32150	Haloacid dehalogenase-like hydrolase (HAD) superfamily protein(AT2G32150)	1.523809252	1.763348278	0.646587019
At4g11900	S-locus lectin protein kinase family protein(AT4G11900)	1.524485278	0.533271921	0.783592523
At3g05180	GDSL-like Lipase/Acylhydrolase superfamily protein(AT3G05180)	1.524577291	0.30750303	0.718618962
At1g70370	polygalacturonase 2(PG2)	1.525115257	0.088923944	0.179412514
At2g01450	MAP kinase 17(MPK17)	1.525251756	1.594556011	0.91533956
At3g16290	AAA-type ATPase family protein(EMB2083)	1.525636412	0.472458718	0.865259539
At5g51560	Leucine-rich repeat protein kinase family protein(AT5G51560)	1.525681458	0.251398421	0.271259105
At1g64670	alpha/beta-Hydrolases superfamily protein(BDG1)	1.526234191	0.283343174	0.406883874
At5g36710	transmembrane protein(AT5G36710)	1.526834093	0.549569879	0.453106135
At1g77900	unknown protein	1.527040882	1.788978859	1.420664545
At2g46225	ABI-1-like 1(ABIL1)	1.527146434	0.624354029	0.567854433

At4g14365	hypothetical protein(XBAT34)	1.528185901	0.584721887	0.490896544
At1g61140	SNF2 domain-containing protein / helicase domain-containing protein / zinc finger prot	1.528460226	1.555372467	1.468705235
At5g65280	GCR2-like 1(GCL1)	1.52868225	0.501089352	0.41276128
At5g53880	hypothetical protein(AT5G53880)	1.528897897	0.231806449	0.65496017
At4g35600	Protein kinase superfamily protein(CST)	1.528942766	1.8117674	0.955146309
At5g51200	nuclear pore complex protein (DUF3414)(EMB3142)	1.529438012	0.442638784	0.550433144
At2g25250	serine/arginine repetitive matrix-like protein(AT2G25250)	1.529444688	0.508550982	0.48402341
At1g17140	interactor of constitutive active rops 1(ICR1)	1.530118867	0.658432986	0.573939481
At5g62570	Calmodulin binding protein-like protein(AT5G62570)	1.53090713	1.066947941	0.593184323
At4g26690	PLC-like phosphodiesterase family protein(SHV3)	1.531299601	0.440143024	0.768794305
At1g07510	FTSH protease 10(ftsh10)	1.532880504	0.623828167	1.033369731
At4g39450	hypothetical protein	1.532883661	2.123734369	2.204522087
At2g43530	scorpion toxin-like knottin superfamily protein(AT2G43530)	1.535224227	0.218918819	0.466444837
At3g23080	Polyketide cyclase/dehydrase and lipid transport superfamily protein(AT3G23080)	1.535836785	3.256551591	1.665716799
At4g20930	6-phosphogluconate dehydrogenase family protein(AT4G20930)	1.536045135	5.085289171	2.266759352
At3g10720	Plant invertase/pectin methylesterase inhibitor superfamily(AT3G10720)	1.53625352	0.247953075	0.160101036
At1g68780	RNI-like superfamily protein(AT1G68780)	1.537696335	0.563287224	0.522933565
At2g28470	beta-galactosidase 8(BGAL8)	1.537697771	0.252346769	0.371381877
At4g19510	Disease resistance protein (TIR-NBS-LRR class)(AT4G19510)	1.539936242	1.640462826	1.422574974
At1g33560	Disease resistance protein (CC-NBS-LRR class) family(ADR1)	1.539950318	1.629100392	1.914099006
At1g02660	alpha/beta-Hydrolases superfamily protein(AT1G02660)	1.54065827	2.428983588	1.170795804
At3g43610	Spc97 / Spc98 family of spindle pole body (SBP) component(AT3G43610)	1.540799446	0.408869261	0.624725544
At1g14700	purple acid phosphatase 3(PAP3)	1.540861999	0.265669502	0.367117871
At3g26230	cytochrome P450, family 71, subfamily B, polypeptide 24(CYP71B24)	1.541575784	10.27832416	7.552503844
At5g50420	O-fucosyltransferase family protein(AT5G50420)	1.54220286	0.246268534	0.671742903
At1g52400	beta glucosidase 18(BGLU18)	1.543386361	0.121112249	0.050080104
At3g25600	Calcium-binding EF-hand family protein(AT3G25600)	1.543903959	0.416311864	0.530529293
At1g33240	GT-2-like 1(GTL1)	1.544508887	2.696523994	1.190876162
At3g19850	Phototropic-responsive NPH3 family protein(AT3G19850)	1.544902431	7.101624818	2.835135496
At3g20590	Late embryogenesis abundant (LEA) hydroxyproline-rich glycoprotein family(AT3G205	1.545792778	0.216325182	0.51966385
At3g11120	Ribosomal protein L41 family(AT3G11120)	1.545912689	0.304810632	0.581342384
At2g30010	TRICHOME BIREFRINGENCE-LIKE 45(TBL45)	1.546902519	0.244476297	0.234145734

At5g06190	transmembrane protein(AT5G06190)	1.547053834	1.599866818	2.295938398
At5g02200	far-red-elongated hypocotyl1-like protein(FHL)	1.54933954	1.715909254	0.867409097
At4g18030	S-adenosyl-L-methionine-dependent methyltransferases superfamily protein(AT4G18(	1.550499812	0.249066136	0.558463971
At2g46450	cyclic nucleotide-gated channel 12(CNGC12)	1.551176411	0.813879559	2.244652169
At5g37540	Eukaryotic aspartyl protease family protein(AT5G37540)	1.552214358	2.413292438	1.505053117
At5g25930	kinase family with leucine-rich repeat domain-containing protein(AT5G25930)	1.552528887	0.446220141	0.523962545
At5g53130	cyclic nucleotide gated channel 1(CNGC1)	1.552787589	1.683181577	1.452317475
At2g29510	hypothetical protein (DUF3527)(AT2G29510)	1.554722673	0.532796208	0.787653295
At5g05480	Peptide-N4-(N-acetyl-beta-glucosaminyl)asparagine amidase A protein(AT5G05480)	1.556026459	5.885917602	2.664800634
At1g02390	glycerol-3-phosphate acyltransferase 2(GPAT2)	1.55976544	1.577330317	2.288692299
At1g30810	Transcription factor jumonji (jmj) family protein / zinc finger (C5HC2 type) family protei	1.56236974	2.117147781	1.431910985
At2g40610	expansin A8(EXPA8)	1.563794471	0.31299845	0.122212677
At1g24140	Matrixin family protein(AT1G24140)	1.564593394	0.553220287	0.708880839
At1g63090	phloem protein 2-A11(PP2-A11)	1.565098412	1.606598212	2.718122858
At1g13750	Purple acid phosphatases superfamily protein(AT1G13750)	1.565366537	1.618023907	2.516712213
At5g62630	hipl2 protein precursor(HIPL2)	1.565502194	2.636388093	3.861844878
At4g34220	Leucine-rich repeat protein kinase family protein(AT4G34220)	1.565555357	0.356258489	0.369090964
At3g42660	transducin family protein / WD-40 repeat family protein(AT3G42660)	1.565766014	0.630903267	0.627062862
At3g14940	phosphoenolpyruvate carboxylase 3(PPC3)	1.567256969	0.695515607	0.652934578
At1g69790	Protein kinase superfamily protein(AT1G69790)	1.568185285	2.175343581	2.664768221
At3g47560	alpha/beta-Hydrolases superfamily protein(AT3G47560)	1.568422862	0.403429863	0.559937079
At1g07000	exocyst subunit exo70 family protein B2(EXO70B2)	1.569011626	2.237195161	0.921438601
At1g20510	OPC-8:0 CoA ligase1(OPCL1)	1.569771219	0.234481155	0.279404533
At2g31880	Leucine-rich repeat protein kinase family protein(SOBIR1)	1.570191805	0.972939843	0.631026099
At1g72930	toll/interleukin-1 receptor-like protein(TIR)	1.571407514	0.125558249	1.47806104
At5g23210	serine carboxypeptidase-like 34(SCPL34)	1.573144552	2.046097836	0.8140332
At1g23710	hypothetical protein (DUF1645)(AT1G23710)	1.574818807	1.690566799	1.397403079
At3g15350	Core-2/I-branching beta-1,6-N-acetylglucosaminyltransferase family protein(AT3G153	1.575329817	0.427652003	0.970027538
At5g25440	Protein kinase superfamily protein(AT5G25440)	1.582179995	1.947540996	0.972839131
At2g25200	hypothetical protein (DUF868)(AT2G25200)	1.582737082	4.996486315	2.270034091
At5g10695	methionyl-tRNA synthetase(AT5G10695)	1.58527244	0.742478759	0.495186809
At3g28270	transmembrane protein, putative (DUF677)(AT3G28270)	1.586655683	0.463366229	2.185109659

At3g15530	S-adenosyl-L-methionine-dependent methyltransferases superfamily protein(AT3G155	1.587073233	0.496288496	0.516660982
At2g41090	Calcium-binding EF-hand family protein(AT2G41090)	1.588067239	0.130966583	0.759253573
At1g79560	FTSH protease 12(FTSH12)	1.590818198	0.257901016	0.638898652
At2g40670	response regulator 16(RR16)	1.591059403	1.684811679	1.860887702
At3g02110	serine carboxypeptidase-like 25(scpl25)	1.595675142	0.37770457	0.433308038
At1g10850	Leucine-rich repeat protein kinase family protein(AT1G10850)	1.596759137	0.583072911	0.664466583
At1g75130	cytochrome P450, family 721, subfamily A, polypeptide 1(CYP721A1)	1.6005642	2.421485035	1.629634636
At1g23080	Auxin efflux carrier family protein(PIN7)	1.601629617	0.274260518	0.221755485
At1g14030	Rubisco methyltransferase family protein(LSMT-L)	1.601691989	0.532670515	1.065325059
At3g04640	glycine-rich protein(AT3G04640)	1.601693564	0.231863624	0.259321271
At2g23130	arabinogalactan protein 17(AGP17)	1.603168413	0.74989648	1.803292106
At4g03390	STRUBBELIG-receptor family 3(SRF3)	1.603170206	1.716191811	1.594517466
At4g22780	ACT domain repeat 7(ACR7)	1.604244216	0.517105628	0.354331641
At2g34510	choice-of-anchor C domain protein, putative (Protein of unknown function, DUF642)(A	1.606557064	0.558136148	0.523145279
At2g37130	Peroxidase superfamily protein(AT2G37130)	1.606784866	121.4637375	59.23397894
At3g14840	Leucine-rich repeat transmembrane protein kinase(AT3G14840)	1.608560355	0.489481475	0.705713574
At3g07990	serine carboxypeptidase-like 27(SCPL27)	1.608831234	0.560553039	0.952367864
At2g13790	somatic embryogenesis receptor-like kinase 4(SERK4)	1.608974111	0.862631929	0.56055502
At1g11545	xyloglucan endotransglucosylase/hydrolase 8(XTH8)	1.613041515	0.301348138	0.257362548
At1g69570	Dof-type zinc finger DNA-binding family protein(AT1G69570)	1.613602745	3.673081885	1.639856425
At4g37550	Acetamidase/Formamidase family protein(AT4G37550)	1.613750523	1.576167401	1.735930666
At5g58960	glucose-6-phosphate isomerase, putative (DUF641)(GIL1)	1.615388425	1.863851406	1.230283928
At2g40550	E2F target protein 1 (ETG1)(ETG1)	1.615634384	0.525534049	0.471088591
At5g02230	Haloacid dehalogenase-like hydrolase (HAD) superfamily protein(AT5G02230)	1.616299596	1.48688038	3.662757934
At5g01670	NAD(P)-linked oxidoreductase superfamily protein(AT5G01670)	1.622231488	3.425601133	2.487245615
At1g50240	kinase family with ARM repeat domain-containing protein(FU)	1.62500607	0.774531128	0.651939509
At3g06850	2-oxoacid dehydrogenases acyltransferase family protein(BCE2)	1.628658134	13.48333217	3.187943928
At5g67450	zinc-finger protein 1(ZF1)	1.62933132	3.080397956	2.027772453
At2g19620	N-MYC downregulated-like 3(NDL3)	1.630617125	0.578342847	0.589466123
At5g18360	Disease resistance protein (TIR-NBS-LRR class) family(AT5G18360)	1.630692491	1.655895129	1.247560452
At4g37450	arabinogalactan protein 18(AGP18)	1.633805391	0.420616543	0.453334517
At4g11280	1-aminocyclopropane-1-carboxylic acid (acc) synthase 6(ACS6)	1.638997861	0.343821805	0.370926354

At2g05920	Subtilase family protein(AT2G05920)	1.639723663	0.178326482	0.619552443
At1g34750	Protein phosphatase 2C family protein(AT1G34750)	1.642966426	2.866301349	2.352451628
At1g14280	phytochrome kinase substrate 2(PKS2)	1.647266462	1.675347468	0.699066978
At3g13980	SKI/DACH domain protein(AT3G13980)	1.652896695	0.517479597	0.463431357
At3g15940	UDP-Glycosyltransferase superfamily protein(AT3G15940)	1.652954282	0.612648969	1.029177706
At2g17550	RB1-inducible coiled-coil protein(TRM26)	1.6558838	7.07519142	3.058212183
At1g79380	Ca(2)-dependent phospholipid-binding protein (Copine) family(RGLG4)	1.657160477	1.790488227	0.954997055
At5g50570	Squamosa promoter-binding protein-like (SBP domain) transcription factor family prote	1.657923542	0.186234304	0.138410842
At3g25500	formin homology 1(AFH1)	1.658913915	0.385385853	0.842082281
At1g15290	Tetratricopeptide repeat (TPR)-like superfamily protein(AT1G15290)	1.659232396	0.564580715	1.382476352
At3g10525	LOSS OF GIANT CELLS FROM ORGANS(LGO)	1.660061977	2.354590879	2.58679479
At1g17380	jasmonate-zim-domain protein 5(JAZ5)	1.663983823	0.099293817	0.096135901
At1g68585	hypothetical protein(AT1G68585)	1.669272242	2.705858332	1.816019155
At1g56720	Protein kinase superfamily protein(AT1G56720)	1.672484605	0.529524734	0.689358126
At4g33000	calcineurin B-like protein 10(CBL10)	1.673720174	0.400411894	0.868963826
At3g11700	FASCICLIN-like arabinogalactan protein 18 precursor(FLA18)	1.67404822	0.220792757	0.362990136
At3g46600	GRAS family transcription factor(AT3G46600)	1.674083648	1.559101902	0.933296389
At1g29430	SAUR-like auxin-responsive protein family(AT1G29430)	1.676559035	0.374113565	0.287827478
At1g57680	plasminogen activator inhibitor(Cand1)	1.676746083	1.802875193	1.587935409
At5g08540	ribosomal RNA small subunit methyltransferase J(AT5G08540)	1.677817682	1.993262968	1.599612757
At1g28480	Thioredoxin superfamily protein(GRX480)	1.680145265	0.408747841	0.420019739
At1g05630	Endonuclease/exonuclease/phosphatase family protein(5PTASE13)	1.685067887	0.516015176	0.513966579
At5g39670	Calcium-binding EF-hand family protein(AT5G39670)	1.686975794	2.422718101	3.639830051
At4g39900	adenine deaminase(AT4G39900)	1.687498535	0.562475508	0.467078826
At3g21230	4-coumarate:CoA ligase 5(4CL5)	1.687614505	1.070001892	2.166171385
At5g13980	Glycosyl hydrolase family 38 protein(AT5G13980)	1.689301255	0.52692746	0.602614443
At1g73540	nudix hydrolase homolog 21(NUDT21)	1.69112147	0.299304446	0.250530413
At2g26170	cytochrome P450, family 711, subfamily A, polypeptide 1(CYP711A1)	1.692644185	6.396071662	3.236571063
At3g57330	autoinhibited Ca2+-ATPase 11(ACA11)	1.693987355	1.638601366	1.261434514
At3g44720	arogenate dehydratase 4(ADT4)	1.695446684	0.492412867	1.002141307
At3g51450	Calcium-dependent phosphotriesterase superfamily protein(AT3G51450)	1.696394729	0.118439405	0.130404483
At1g76540	cyclin-dependent kinase B2;1(CDKB2;1)	1.700424251	0.773505237	0.647895506

At3g27560	Protein kinase superfamily protein(ATN1)	1.702813679	2.785287019	1.875235332
At1g80840	WRKY DNA-binding protein 40(WRKY40)	1.703361506	0.164414447	0.138114348
At5g16910	cellulose-synthase like D2(CSLD2)	1.703890403	4.770312534	4.028783025
At5g49280	hydroxyproline-rich glycoprotein family protein(AT5G49280)	1.706806499	0.56856445	0.461376581
At1g19770	purine permease 14(PUP14)	1.711651526	0.627905958	0.493922949
At4g27710	cytochrome P450, family 709, subfamily B, polypeptide 3(CYP709B3)	1.712353209	1.219349143	1.850135252
At1g66350	RGA-like 1(RGL1)	1.713319947	0.718897759	0.446095552
At4g01330	Protein kinase superfamily protein(AT4G01330)	1.717137792	4.648153926	1.523055831
At5g37770	EF hand calcium-binding protein family(TCH2)	1.72151418	0.198779035	0.428667893
At1g18620	LONGIFOLIA protein(TRM3)	1.722510283	1.621056096	0.405473868
At2g41120	DUF309 domain protein(AT2G41120)	1.722899859	1.649071777	2.73723323
At5g17350	hypothetical protein(AT5G17350)	1.723456178	0.250154468	0.2916149
At5g56850	hypothetical protein(AT5G56850)	1.734984836	0.450970547	1.215639859
At4g30440	UDP-D-glucuronate 4-epimerase 1(GAE1)	1.737637856	0.353001223	0.387534303
At4g25260	Plant invertase/pectin methylesterase inhibitor superfamily protein(AT4G25260)	1.740826682	1.193178177	0.534753774
At4g14550	indole-3-acetic acid inducible 14(IAA14)	1.74932344	0.98227364	0.465122914
At1g33940	Serine/Threonine-kinase ULK4-like protein(AT1G33940)	1.750491476	0.472959662	0.588584086
At2g34930	disease resistance family protein / LRR family protein(AT2G34930)	1.753844511	0.197495127	0.125471262
At1g72970	Glucose-methanol-choline (GMC) oxidoreductase family protein(HTH)	1.754599982	0.11447801	0.168303807
At5g38700	cotton fiber protein(AT5G38700)	1.758885588	0.554283652	0.620755868
At5g64570	beta-D-xylosidase 4(XYL4)	1.759709901	14.11529452	12.9179717
At1g61667	serine protease, putative (Protein of unknown function, DUF538)(AT1G61667)	1.762743239	1.132704786	1.646248098
At3g61640	arabinogalactan protein 20(AGP20)	1.769460473	0.400972301	0.504307336
At2g43290	Calcium-binding EF-hand family protein(MSS3)	1.772054374	0.412057743	0.801105015
At1g44100	amino acid permease 5(AAP5)	1.775459019	2.426142994	3.382957256
At3g08720	serine/threonine protein kinase 2(S6K2)	1.777565767	2.185222709	1.194588901
At1g11960	ERD (early-responsive to dehydration stress) family protein(AT1G11960)	1.779283596	0.355315153	0.335202606
At3g46540	ENTH/VHS family protein(AT3G46540)	1.783893019	0.862071834	2.668848124
At5g19240	Glycoprotein membrane precursor GPI-anchored(AT5G19240)	1.786074614	0.115459161	0.316021271
At2g05940	Protein kinase superfamily protein(RIPK)	1.786614209	0.337353137	0.821971073
At1g63240	hypothetical protein(AT1G63240)	1.788026244	0.583128353	0.591540377
At3g25400	dCTP pyrophosphatase-like protein(AT3G25400)	1.793427212	0.476247272	0.508076442

At2g44230	hypothetical protein (DUF946)(AT2G44230)	1.795802404	0.362949308	0.542395424
At4g18010	myo-inositol polyphosphate 5-phosphatase 2(IP5PII)	1.797709103	2.243048593	2.19042357
At5g02940	ion channel POLLUX-like protein, putative (DUF1012)(AT5G02940)	1.798423592	0.164337604	0.349457661
At3g13560	O-Glycosyl hydrolases family 17 protein(AT3G13560)	1.798953628	0.233018032	0.332546199
At1g17990	FMN-linked oxidoreductases superfamily protein(AT1G17990)	1.800091114	0.81056113	0.401759205
At1g55330	arabinogalactan protein 21(AGP21)	1.800311218	0.245081179	0.356695758
At5g10560	Glycosyl hydrolase family protein(AT5G10560)	1.800717881	0.470390282	0.939382566
At5g05300	hypothetical protein(AT5G05300)	1.80681435	0.333017692	0.324941957
At4g02850	phenazine biosynthesis PhzC/PhzF family protein(AT4G02850)	1.811824883	0.171331715	0.14936936
At4g35060	Heavy metal transport/detoxification superfamily protein(HIPP25)	1.812097555	1.868633363	6.591734114
At1g48320	Thioesterase superfamily protein(DHNAT1)	1.813912528	4.514242421	2.897971495
At1g70790	Calcium-dependent lipid-binding (CaLB domain) family protein(AT1G70790)	1.820886886	0.990640926	1.679959086
At1g58250	HYPERSENSITIVE TO PI STARVATION 4(SAB)	1.823663578	2.01462113	1.935060287
At4g34150	Calcium-dependent lipid-binding (CaLB domain) family protein(AT4G34150)	1.824818492	0.398517661	0.532818243
At5g54490	pinoid-binding protein 1(PBP1)	1.825518289	0.120453553	0.253725526
At2g12400	plasma membrane fusion protein(AT2G12400)	1.826684535	0.23085375	0.447621967
At1g28370	ERF domain protein 11(ERF11)	1.827395132	0.129015944	0.16995178
At1g20823	RING/U-box superfamily protein(AT1G20823)	1.828588962	0.387305912	0.329698689
At1g34760	general regulatory factor 11(GRF11)	1.844528671	6.138331425	4.635105404
At1g62540	flavin-monooxygenase glucosinolate S-oxygenase 2(FMO GS-OX2)	1.850708527	0.341619419	0.416688485
At5g64850	sorbin/SH3 domain protein(AT5G64850)	1.850932962	0.365026822	0.826461069
At3g10930	hypothetical protein(AT3G10930)	1.851182396	0.31007592	0.36224093
At3g48090	alpha/beta-Hydrolases superfamily protein(EDS1)	1.851695948	0.471909822	0.620198753
At1g59930	MADS-box family protein(AT1G59930)	1.852183433	1.205264901	2.259354826
At5g41100	hydroxyproline-rich glycoprotein family protein(AT5G41100)	1.854349174	0.654575439	0.54790802
At1g67530	ARM repeat superfamily protein(AT1G67530)	1.854599208	2.281433515	1.609839219
At1g80820	cinnamoyl coa reductase(CCR2)	1.859472615	0.930835819	3.260221431
At2g30930	hypothetical protein(AT2G30930)	1.859792123	2.981960058	4.22407277
At3g55980	salt-inducible zinc finger 1(SZF1)	1.869578746	0.326286426	0.332151235
At5g16250	transmembrane protein(AT5G16250)	1.874586833	0.384182611	0.320949795
At2g32100	ovate family protein 16(OFP16)	1.885230208	0.481475534	0.472391441
At5g22630	arogenate dehydratase 5(ADT5)	1.889268188	0.172335149	0.228253738

At2g03240	EXS (ERD1/XPR1/SYG1) family protein(AT2G03240)	1.891207459	0.94900407	0.664011135
At3g06770	Pectin lyase-like superfamily protein(AT3G06770)	1.89802669	0.463668876	0.457665973
At2g27690	cytochrome P450, family 94, subfamily C, polypeptide 1(CYP94C1)	1.913194152	0.285139299	0.254594574
At3g52450	plant U-box 22(PUB22)	1.918926021	3.128946566	1.709195335
At3g04290	Li-tolerant lipase 1(LTL1)	1.920036842	0.04703691	0.044428647
At4g35985	Senescence/dehydration-associated protein-like protein(AT4G35985)	1.920385499	0.531698137	0.444075052
At3g47960	Major facilitator superfamily protein(GTR1)	1.925453533	0.859085747	2.348779721
At2g40270	Protein kinase family protein(AT2G40270)	1.926401083	1.99094822	1.451340581
At1g18740	ROH1, putative (DUF793)(AT1G18740)	1.931699004	0.347498927	0.420269675
At2g33580	Protein kinase superfamily protein(LYK5)	1.933295505	0.338871528	0.609129388
At4g34760	SAUR-like auxin-responsive protein family(AT4G34760)	1.933819636	0.260822901	0.292866636
At1g16370	organic cation/carnitine transporter 6(OCT6)	1.934175081	0.38171448	0.244487214
At1g74450	BPS1-like protein (DUF793)(AT1G74450)	1.940459049	0.281409695	0.294447195
At5g08350	GRAM domain-containing protein / ABA-responsive protein-like protein(AT5G08350)	1.941913813	6.423578229	3.253590368
At4g36030	armadillo repeat only 3(ARO3)	1.942991419	0.700679374	0.645037348
At2g17040	NAC domain containing protein 36(NAC036)	1.946681277	0.531643016	0.870459378
At1g13700	6-phosphogluconolactonase 1(PGL1)	1.948678319	9.376053086	7.237841652
At1g19380	sugar, putative (DUF1195)(AT1G19380)	1.949711878	0.471939367	0.352154325
At4g25830	Uncharacterized protein family (UPF0497)(AT4G25830)	1.954746566	0.569753081	0.550731154
At5g11070	hypothetical protein(AT5G11070)	1.959656308	0.366506383	0.501786704
At3g25180	cytochrome P450, family 82, subfamily G, polypeptide 1(CYP82G1)	1.96002005	0.397585227	0.272569225
At1g66180	Eukaryotic aspartyl protease family protein(AT1G66180)	1.962490929	0.239576015	0.231860072
At5g54190	protochlorophyllide oxidoreductase A(PORA)	1.967458401	0.136304078	0.283657437
At1g27460	no pollen germination related 1(NPGR1)	1.96864776	0.423101784	0.817825769
At1g74440	ER membrane protein, putative (DUF962)(AT1G74440)	1.987517238	0.76431582	0.515696641
At1g30040	gibberellin 2-oxidase(GA2OX2)	1.994705897	2.450757445	1.297866946
At4g22010	SKU5 similar 4(sks4)	1.999239794	0.255057616	0.707270879
At5g24590	TCV-interacting protein(TIP)	2.005702964	1.014824504	0.625138726
At2g39650	cruciferin (DUF506)(AT2G39650)	2.005839572	0.580308348	0.677386953
At3g62720	xylosyltransferase 1(XT1)	2.00974809	0.383329302	0.457063686
At2g35880	TPX2 (targeting protein for Xklp2) protein family(AT2G35880)	2.01298603	0.605694672	0.52276277
At5g15350	early nodulin-like protein 17(ENODL17)	2.017550342	0.504173796	1.235468901

At1g11670	MATE efflux family protein(AT1G11670)	2.018162045	0.41170325	0.324974873
At2g24600	Ankyrin repeat family protein(AT2G24600)	2.021016169	0.2078773	0.222163101
At5g04720	ADR1-like 2(ADR1-L2)	2.023761049	3.824837281	1.890889265
At3g56710	sigma factor binding protein 1(SIB1)	2.024896305	0.267103505	0.424673397
At1g23480	cellulose synthase-like A3(CSLA03)	2.029993833	0.682527369	0.597274114
At3g59080	Eukaryotic aspartyl protease family protein(AT3G59080)	2.03041273	0.507347464	0.494424758
At1g10020	formin-like protein (DUF1005)(AT1G10020)	2.035686309	1.666418687	1.339669667
At4g31840	early nodulin-like protein 15(ENODL15)	2.057462438	0.275786052	0.247259647
At1g73080	PEP1 receptor 1(PEPR1)	2.05765206	0.473589861	0.310165377
At1g24070	cellulose synthase-like A10(CSLA10)	2.063460997	0.408397612	0.364658433
At1g78970	lupeol synthase 1(LUP1)	2.082136196	0.348623694	0.160937192
At3g57450	hypothetical protein(AT3G57450)	2.091162437	0.347997836	0.53413508
At1g35350	EXS (ERD1/XPR1/SYG1) family protein(AT1G35350)	2.096370495	2.077403978	2.170190349
At5g47910	respiratory burst oxidase homologue D(RBOHD)	2.100090925	0.293191883	0.516780305
At4g23180	cysteine-rich RLK (RECEPTOR-like protein kinase) 10(CRK10)	2.103063293	0.225223118	0.250246723
At1g50740	Transmembrane proteins 14C(AT1G50740)	2.104458915	0.889320875	0.603281001
At1g49840	glutamyl-tRNA (Gln) amidotransferase subunit A (DUF620)(AT1G49840)	2.118098618	0.813772132	0.585271652
At3g45640	mitogen-activated protein kinase 3(MPK3)	2.119994268	0.897563741	0.628879789
At3g22240	cysteine-rich/transmembrane domain PCC1-like protein(AT3G22240)	2.121570634	0.106888394	0.761316054
At3g22231	pathogen and circadian controlled 1(PCC1)	2.128999078	0.020138574	0.529282086
At3g23890	topoisomerase II(TOPII)	2.131180248	0.673709264	0.665991149
At1g54820	Protein kinase superfamily protein(AT1G54820)	2.134887571	0.178087059	0.288494379
At4g17490	ethylene responsive element binding factor 6(ERF6)	2.135572757	0.111002796	0.179597138
At1g21270	wall-associated kinase 2(WAK2)	2.140736889	0.341809164	0.695155067
At1g67470	Protein kinase superfamily protein(AT1G67470)	2.140772579	0.531358809	0.488215971
At3g50060	myb domain protein 77(MYB77)	2.148697804	0.223942749	0.430100806
At1g66160	CYS, MET, PRO, and GLY protein 1(CMPG1)	2.162838423	1.769371194	1.772097453
At1g02205	Fatty acid hydroxylase superfamily(CER1)	2.167660305	1.275157896	3.956043393
At3g28180	Cellulose-synthase-like C4(CSLC04)	2.181255406	0.189573336	0.220855507
At5g63140	purple acid phosphatase 29(PAP29)	2.183739622	0.580537532	0.608622427
At5g65470	O-fucosyltransferase family protein(AT5G65470)	2.184575125	0.513838799	0.77417178
At4g34410	redox responsive transcription factor 1(RRTF1)	2.187951061	0.196575859	0.211607742

At1g68410	Protein phosphatase 2C family protein(AT1G68410)	2.206770724	1.5369984	2.035846353
At5g45340	cytochrome P450, family 707, subfamily A, polypeptide 3(CYP707A3)	2.210135173	0.254751471	0.329141485
At1g52290	Protein kinase superfamily protein(PERK15)	2.215615165	0.235822869	0.323723092
At5g50915	basic helix-loop-helix (bHLH) DNA-binding superfamily protein(AT5G50915)	2.216477599	0.597996823	1.463130015
At1g44830	Integrase-type DNA-binding superfamily protein(AT1G44830)	2.219910498	0.600749265	0.54095277
At3g09870	SAUR-like auxin-responsive protein family(AT3G09870)	2.230264663	0.353999666	0.355882441
At4g38400	expansin-like A2(EXLA2)	2.235188496	0.217322186	0.213909685
At4g29780	nuclease(AT4G29780)	2.245283953	0.206553671	0.240226803
At3g04210	Disease resistance protein (TIR-NBS class)(AT3G04210)	2.258798406	0.103646929	0.232972224
At4g27280	Calcium-binding EF-hand family protein(AT4G27280)	2.266404114	0.236214637	0.340620051
At5g48450	SKU5 similar 3(sks3)	2.276321591	0.402575186	0.461150637
At2g26190	calmodulin-binding family protein(AT2G26190)	2.281147467	0.544721894	0.520698895
At4g39510	cytochrome P450, family 96, subfamily A, polypeptide 12(CYP96A12)	2.292053881	0.229978696	0.269622187
At3g05490	ralf-like 22(RALFL22)	2.293472777	0.516972014	0.742910293
At5g48490	Bifunctional inhibitor/lipid-transfer protein/seed storage 2S albumin superfamily protein	2.295026604	0.072438037	0.112595548
At3g49530	NAC domain containing protein 62(NAC062)	2.302926798	0.579230894	0.621913467
At1g03870	FASCICLIN-like arabinoogalactan 9(FLA9)	2.304506322	0.469576372	0.359949403
At3g50930	cytochrome BC1 synthesi(BCS1)	2.333540603	0.130170096	0.237040474
At4g04540	cysteine-rich RLK (RECEPTOR-like protein kinase) 39(CRK39)	2.346133338	0.510944056	0.890522442
At2g14290	LL-diaminopimelate protein (DUF295)(AT2G14290)	2.352389747	0.73280494	0.552599466
At4g18340	Glycosyl hydrolase superfamily protein(AT4G18340)	2.353996753	0.708539005	0.378231386
At5g62165	AGAMOUS-like 42(AGL42)	2.36180648	1.448749417	0.648871789
At1g66090	Disease resistance protein (TIR-NBS class)(AT1G66090)	2.371227873	0.441766827	0.506887837
At1g17620	Late embryogenesis abundant (LEA) hydroxyproline-rich glycoprotein family(AT1G176	2.382108807	0.525479718	0.688256532
At1g27770	autoinhibited Ca2+-ATPase 1(ACA1)	2.405715637	0.850307883	0.659466506
At1g52830	indole-3-acetic acid 6(IAA6)	2.413515344	0.266333067	0.246925543
At1g11050	Protein kinase superfamily protein(AT1G11050)	2.434921316	1.062805838	1.527481939
At2g45340	Leucine-rich repeat protein kinase family protein(AT2G45340)	2.441430723	3.505085755	3.533321257
At4g25390	Protein kinase superfamily protein(AT4G25390)	2.442562884	5.166067958	2.9617726
At2g01180	phosphatidic acid phosphatase 1(PAP1)	2.448021502	0.435316414	0.581612071
At5g03120	transmembrane protein(AT5G03120)	2.479349041	0.663245514	0.585571541
At4g24380	dihydrofolate reductase(AT4G24380)	2.484277943	0.310333131	0.459597487

At1g06080	delta 9 desaturase 1(ADS1)	2.493637857	0.203771536	0.184146905
At2g42760	DUF1685 family protein(AT2G42760)	2.505643888	0.329306715	0.331179878
At5g66210	calcium-dependent protein kinase 28(CPK28)	2.52185145	0.569735569	0.395869111
At5g41740	Disease resistance protein (TIR-NBS-LRR class) family(AT5G41740)	2.531313077	0.706980164	0.549689353
At4g30290	xyloglucan endotransglucosylase/hydrolase 19(XTH19)	2.534355448	0.114550823	1.562031958
At4g02200	Drought-responsive family protein(AT4G02200)	2.53643156	0.603634862	0.510234569
At3g59310	solute carrier family 35 protein (DUF914)(AT3G59310)	2.539283161	0.251805611	0.341380098
At4g01950	glycerol-3-phosphate acyltransferase 3(GPAT3)	2.561728923	0.246676826	0.336709701
At5g04530	3-ketoacyl-CoA synthase 19(KCS19)	2.60011234	0.627970887	0.618426822
At1g72520	PLAT/LH2 domain-containing lipoxygenase family protein(LOX4)	2.603505373	0.201980166	0.180251534
At2g44840	ethylene-responsive element binding factor 13(ERF13)	2.616166264	0.124011594	0.092537374
At5g45670	GDSL-like Lipase/Acylhydrolase superfamily protein(AT5G45670)	2.650863377	0.137424542	0.133763659
At1g19960	transcription factor(AT1G19960)	2.692080785	0.288775205	1.610133711
At3g54000	TIP41-like protein(AT3G54000)	2.749520353	0.733290231	0.648107953
At3g19970	alpha/beta-Hydrolases superfamily protein(AT3G19970)	2.767630413	0.525818435	0.584038983
At2g35710	Nucleotide-diphospho-sugar transferases superfamily protein(PGSIP7)	2.791822911	0.455067303	0.507700952
At2g44500	O-fucosyltransferase family protein(AT2G44500)	2.834462099	0.474361878	0.503834785
At2g17660	RPM1-interacting protein 4 (RIN4) family protein(AT2G17660)	2.852471593	0.551361623	0.73587163
At5g51190	Integrase-type DNA-binding superfamily protein(AT5G51190)	2.866664904	0.155535995	0.276114391
At1g01560	MAP kinase 11(MPK11)	2.871960935	0.299177576	0.465171116
At1g52000	Mannose-binding lectin superfamily protein(AT1G52000)	3.030040353	0.218429493	0.491443354
At3g58120	Basic-leucine zipper (bZIP) transcription factor family protein(BZIP61)	3.035269605	0.525474923	0.39983999
At5g52760	Copper transport protein family(AT5G52760)	3.052311486	0.358307268	1.059103298
At4g17615	calcineurin B-like protein 1(CBL1)	3.06715409	0.455128045	0.475805728
At4g09030	arabinogalactan protein 10(AGP10)	3.072066956	1.986488062	6.272643502
At1g12610	Integrase-type DNA-binding superfamily protein(DDF1)	3.108456153	0.506112242	0.54653533
At4g27654	transmembrane protein(AT4G27654)	3.192249139	0.194735857	0.218500322
At2g17230	EXORDIUM like 5(EXL5)	3.229333395	0.339818041	1.028612775
At2g14560	LURP-one-like protein (DUF567)(LURP1)	3.246001382	0.179506637	3.734525022
At2g34600	jasmonate-zim-domain protein 7(JAZ7)	3.254039168	0.421738079	0.545929166
At1g74430	myb domain protein 95(MYB95)	3.293615272	0.337993274	0.27329898
At3g54810	Plant-specific GATA-type zinc finger transcription factor family protein(BME3)	3.326953076	0.653004092	1.512981047

At2g35290	hypothetical protein(AT2G35290)	3.356547382	0.268544534	0.325650134
At1g24330	ARM repeat superfamily protein(AT1G24330)	3.510387315	0.597848252	0.782972927
At4g02330	Plant invertase/pectin methylesterase inhibitor superfamily(ATPMEPCRB)	3.523173901	0.24329396	1.182454161
At2g46400	WRKY DNA-binding protein 46(WRKY46)	3.601714108	0.382083187	0.589050311
At1g56510	Disease resistance protein (TIR-NBS-LRR class)(WRR4)	3.74997708	0.271535784	0.479578469
At2g27080	Late embryogenesis abundant (LEA) hydroxyproline-rich glycoprotein family(AT2G270	4.062011319	0.56271845	0.672764507
At1g73800	Expressed protein	4.217943218	0.983526658	1.86574985
At4g03210	xyloglucan endotransglucosylase/hydrolase 9(XTH9)	4.284980414	0.185309663	0.241409234
At1g72920	Toll-Interleukin-Resistance (TIR) domain family protein(AT1G72920)	4.455629276	0.439684489	0.991051794
At5g03350	Legume lectin family protein(AT5G03350)	4.494715693	0.027007529	0.824195309
At4g30280	xyloglucan endotransglucosylase/hydrolase 18(XTH18)	4.504068446	0.517992855	0.741916268
At1g18710	myb domain protein 47(MYB47)	4.912026444	0.456069886	0.446000163
At1g33760	Integrase-type DNA-binding superfamily protein(AT1G33760)	5.331835226	0.441928992	0.450482007
At1g21910	Integrase-type DNA-binding superfamily protein(DREB26)	6.12947644	0.645603204	0.971272412
At4g37240	HTH-type transcriptional regulator(AT4G37240)	6.274363197	0.593814105	0.699952413
At2g40750	WRKY DNA-binding protein 54(WRKY54)	6.570980399	0.369187769	2.202369219
At4g08950	Phosphate-responsive 1 family protein(EXO)	14.76106811	0.623420862	0.902689527

Supplementary Table S2. GO terms that are specifically enriched in At*VIP2* overexpressor plants during *Agrobacterium* transformation

GO term	Description
GO:0019375	galactolipid biosynthetic process
GO:0016036	cellular response to phosphate starvation
GO:0009750	response to fructose
GO:0009744	response to sucrose
GO:0008194	UDP-glycosyltransferase activity

Supplementary Table S3. Motif prediction for down- and up-regulated gene set

Gene set	Sequence	Score	RR
Down-Regulated	mCACGTGk*	784.336	33%
	CGTGkmnn	539.882	2%
	TATAwATA	484.633	6%
	ATATATAT	456.403	1%
	GmCACGTn	381.694	6%
Up-Regulated	ACGCGnnn	578.07	6%
	ACGCGTkT	535.442	4%
	yACGCGnn	531.23	3%
	TATATATA	479.895	18%
	ATwAATwA	473.226	15%

*mCACGTGk contains ACGTG that is a core of ABA Responsive Element (ABRE)

Supplementary Table S4. Distribution of ABRE core motif containing genes

Gene Set	Target occurrences	%	Enrichment	χ2 P-value
Down 0h (537)	266	50	1.54	0.0003
Down 48h (44)	23	49	1.49	0.0007
Down 72h (183)	117	64	1.97	<0.0001
Up 0h (504)	202	41	1.25	0.0889
Up 48h (126)	53	42	1.3	0.0556
Up 72h (100)	37	37	1.13	0.3949

Per cent of ACGTG motifs in background: 32.7
